# Characterizing locus specific chromatin structure and dynamics with correlative conventional and super-resolution imaging in living cells

**DOI:** 10.1093/nar/gkac314

**Published:** 2022-05-07

**Authors:** Dushyant Mehra, Santosh Adhikari, Chiranjib Banerjee, Elias M Puchner

**Affiliations:** School of Physics and Astronomy, University of Minnesota, Minneapolis MN, USA; Department of Physiology and Biomedical Engineering, Mayo Clinic, Rochester MN, USA; School of Physics and Astronomy, University of Minnesota, Minneapolis MN, USA; School of Physics and Astronomy, University of Minnesota, Minneapolis MN, USA; School of Physics and Astronomy, University of Minnesota, Minneapolis MN, USA

## Abstract

The dynamic rearrangement of chromatin is critical for gene regulation, but mapping both the spatial organization of chromatin and its dynamics remains a challenge. Many structural conformations are too small to be resolved via conventional fluorescence microscopy and the long acquisition time of super-resolution photoactivated localization microscopy (PALM) precludes the structural characterization of chromatin below the optical diffraction limit in living cells due to chromatin motion. Here we develop a correlative conventional fluorescence and PALM imaging approach to quantitatively map time-averaged chromatin structure and dynamics below the optical diffraction limit in living cells. By assigning localizations to a locus as it moves, we reliably discriminate between bound and unbound dCas9 molecules, whose mobilities overlap. Our approach accounts for changes in DNA mobility and relates local chromatin motion to larger scale domain movement. In our experimental system, we show that compacted telomeres move faster and have a higher density of bound dCas9 molecules, but the relative motion of those molecules is more restricted than in less compacted telomeres. Correlative conventional and PALM imaging therefore improves the ability to analyze the mobility and time-averaged nanoscopic structural features of locus specific chromatin with single molecule sensitivity and yields unprecedented insights across length and time scales.

## INTRODUCTION

The spatio-temporal organization of chromatin regulates gene expression on various length and timescales. On the smallest length scale of individual nucleosomes (∼10 nm), which is also referred to as the primary structure of chromatin, covalent modification of histone tails regulates the interaction of nucleosomes with DNA and therefore chromatin accessibility (1–3). The secondary structure integrates features of the primary structure in organizations up to ∼100 nm (1–3). The tertiary structure of chromatin comprises larger domains such as enhancer promoter contacts to regulate gene expression (1,3). In addition the regulation of DNA accessibility controls transcription factor binding (4). Even larger chromatin structures such as topologically associated domains can create long range contacts for instance in super-enhancer complexes to activate transcription (2). Many sequencing based methods have been used to indirectly characterize chromatin structure across these length scale (4). However, these methods only provide time-averaged structural information of a population of cells or a structural snapshot of a single cell (2,5). Recent evidence suggests that chromatin compaction, condensation, and accessibility is dynamically regulated at both small and large length scales as well as fast and slow time scales (6). The degree in correlated movement between small chromatin structures and the large chromatin domains they reside in has been recognized as an important feature of nuclear phase condensates, which are hypothesized to help regulate gene expression (6–11). Therefore, studying the nanoscopic structure and dynamics of chromatin across time and length scales is needed to characterize how genome organization affects gene regulation (6,7,12). Here, we present a correlative conventional fluorescence and photoactivated localization microscopy (PALM) approach that overcomes current limitations to study the relation between time-averaged chromatin structure such as the size or compaction of a locus and its dynamics across various time and length scales in living cells.

Recently, CRISPR/dCas9-based fluorescence imaging methods have been developed as a modular approach to map chromatin structure and dynamics. By using programmable guide RNAs (gRNA), fluorophores can be targeted to specific sequences in the genome (13–16). These methods require tens of fluorescent probes to be bound to a locus of interest in order to overcome the background fluorescence from the majority of freely diffusing and searching probes (13,15–18). To reduce the number of gRNAs needed to detect the fluorescence signal of a locus, repetitive RNA aptamers such as MS2 sequences have been appended to gRNAs (19–22). By employing these labeling strategies in conjunction with conventional fluorescence time-lapse imaging, valuable information about the slow and long-term dynamics of loci has been obtained. However, the optical diffraction limit precludes the ability of conventional fluorescence imaging to map the organization of smaller chromatin structures below ∼250 nm. With the advent of photoactivated localization microscopy (PALM), it became possible to track single molecules in living cells (also called single-particle tracking PALM, sptPALM) (23,24) and to resolve intracellular structures in fixed cells with ∼20 nm resolution (25,26). By employing sparse activation of photoactivatable or photoswitchable fluorophores, PALM avoids spatio-temporal overlap of dense emitters and allows for the determination of the precise locations of individual fluorophores by Gaussian fitting. Over time many localizations are then accumulated for linking them to molecular trajectories or for resolving structures below the optical diffraction limit. Recently, CRISPR/dCas9-based DNA labeling has been combined with PALM to monitor chromatin dynamics in living eukaryotic and prokaryotic cells or to obtain structural information such as chromatin compaction or condensation in chemically fixed cells (27–33). However, the long acquisition times required for PALM imaging preclude the ability to obtain both, structural and dynamic information in living cells because the motion of DNA spreads out the localizations of fluorophores bound to a locus along its trajectory. Therefore, it is a challenge to relate chromatin structure and dynamics across length and time scales and to characterize dynamic chromatin rearrangements below the optical diffraction limit (6).

Correlative single molecule and conventional (or stimulated emission depletion, STED) approaches have been previously used to study the nanoscale organization and the dynamics of various biological structures in living cells (34–42). These studies involve dual tagging of the same protein or of interacting proteins with a conventional and a PALM compatible fluorophore. In this way, it has been possible to observe how the protein appended with the PALM tag moves relative to the conventionally tagged protein. These methods have been employed to characterize protein interaction kinetics and to obtain both dynamic and structural information about specific organelles (34–40). Likewise, STED (43–45) and SIM (46,47) can obtain structural and dynamic information in living cells below the optical diffraction limit. However, super-resolved structural information of a specific locus and its overall motion cannot be related to the nanoscopic motion of single fluorophores in living cells, which is key for determining dynamic re-arrangements of chromatin.

Here we develop a correlative conventional fluorescence and PALM imaging approach in living cells to bridge these gaps of current techniques. This approach allows to resolve and quantify time-averaged chromatin structures by correcting for their motion and to simultaneously measure their nanoscopic re-arrangements. In addition, single fluorescent probes are more reliably identified to be associated with a moving locus in order to determine their mobility state. We demonstrate correlative conventional and PALM imaging using the well characterized telomere sequences as a model system. First, we tagged dCas9 and the MS2 coat proteins (MCP) that bind to a modified telomere-targeting gRNA scaffold with a conventional and a spectrally distinct PALM fluorophore. Next, each telomere is tracked with the conventional fluorescence signal while the single molecule localizations are recorded in the PALM channel of the microscope. The trajectory of the locus is then subtracted from the coordinates of the single molecule localizations to correct for motion blurring. As a result, structural parameters such as the time-averaged size of a locus or the density of bound probes can be quantified, which can yield insights into the compaction of chromatin. By determining the location and mobility of a locus relative to the traces of single dCas9/MCP complexes during imaging, dCas9/MCP complexes can be more reliably identified to be bound to a locus. This relative motion of single molecules compared to the locus shows how smaller scale chromatin rearrangements occur within the larger scale chromatin movements. Furthermore, we relate the compaction and condensation of telomere clusters to their local and global chromatin mobility. This study demonstrates that correlative conventional fluorescence and PALM imaging accurately identifies Cas9 molecules bound to a locus and yields quantitative dynamic and time-averaged structural information about specific genomic loci at the nanoscale in living cells.

## MATERIALS AND METHODS

### Plasmid generation

2XMS2 gRNA scaffold and MCP-HaloTag plasmids were obtained from Thoru Pederson through Addgene (20,21). The CMV-S.p.dCas9-VP64 plasmid obtained from Charles Gerbasch through Addgene was used as the backbone vector. A PCR was performed with eGFP using Thermofisher Platinum Taq High Fidelity to add BsteII and AflII restriction enzyme sites on the N-terminus and C-terminus of GFP respectively with the forward primer: AGTCAGCTAGGAGgtgacccaggagctcccaagaaaaagcgcaaggtaggtagttccgtgagcaagggcgaggagctaand reverse primer GCTGATCAGCGGTTTAAACttaagtttacttgtacagctcgtccatgccgag. The purified PCR product and CMV-dCas9-VP64 backbone were digested using NEB BsteII-HF and AflII-HF restriction enzymes, gel purified and ligated using the NEB T4 DNA ligase kit. Ligated plasmids were amplified with DH5α competent cells and purified using Qiagen Miniprep kit. mEos2 (obtained from Davidson Lab) was cloned into a CMV-MCP-YFP plasmid (from Mazhar Adli from Addgene) to replace MCP-YFP (22). A PCR was performed on mEos2 using Thermofisher Platinum Taq High Fidelity to add an BstXI restriction enzyme site to the N terminus of mEos2 and to add a nuclear localization sequence followed by a stop codon and XbaI restriction enzyme site to C terminus of the mEos2 using the forward primer: ggagacccaagcttatgggctacccctacgacgtgcccgactacgccatgagtgcgattaagccagacatg and reverse primer aacttaggccctctagatgcatgttatacctttctcttcttttttggtcgtctggcattg. The PCR product and CMV-MCP-YFP plasmid were digested with BstXI and XbaI restriction enzymes, gel purified, ligated and amplified as before. CRISPR gRNAs sequences targeting telomeres were previously published (13). Repetitive telomere targeting sequences appended with ACCG and AAAC nucleotide overhangs were obtained as individual oligos from IDT with the sequences accgGTTAGGGTTAGGGTTAGGGTTA and aaacTAACCCTAACCCTAACCCTAAC. The oligos were annealed and cloned into a 2xMS2 gRNA scaffold plasmid (obtained from Thoru Pederson through Addgene) using previously described protocols (20,21).

### Cell line culture, transfection and sample preparation

All imaging experiments were performed with Gastrointestinal Stromal Tumor Cells (GIST-T1) provided by Tamas Ordog and Yujiro Hayashi. Cells were cultured in Fluorobrite Media supplemented with 10% Fetal Bovine Serum (FBS), 4 mM L-Glutamine, and 1% Pencillin/Streptomycin to eliminate fluorescence from phenol red. Cells were seeded in No 1.5 8-well plates (IBIDI) at a density of 50000 cells/ml two days prior to imaging. 200 ng of telomere gRNA along with 50 ng of MCP-HaloTag and 50 ng of dCas9-GFP plasmids were transfected into GIST-T1 cells 15–17 hours prior to imaging using the Lipofectamine 3000 and p300 reagent. After transfection, cells were washed twice with serum diluted Fluorobrite media with 1% FBS and 1% Pencillin/Streptomycin. Cells were incubated with 100 nM of PA-JF646 HaloTag dye provided by Bo Huang in serum diluted media for 15 minutes. After incubation cells were washed with serum diluted Fluorobrite media 3 times and placed in a 37°C and 5% CO_2_ incubator for an additional 30 minutes. The washing process was repeated three additional times prior to imaging to remove unbound PA-JF646 dye that is still able to fluoresce. Cells identified as containing gRNA exhibited distinct GFP clusters throughout the nucleus while cells that weren’t transfected with gRNA didn’t contain these distinct nuclear clusters.

### Microscope setup, camera calibration and imaging

The microscope setup and camera calibration that was used for imaging was previously described (48,49). In short, a Nikon inverted microscope (Eclipse Ti-E) equipped with a perfect focus system was used for imaging experiments. All movies were recorded on an Andor iXon 897 Ultra DU-897U electron multiplying charge coupled detector (EMCCD) camera, which was cooled to −70°C and set to an amplifying gain of 30. The 4 excitation lasers (405 nm, 488 nm, 561 nm, 640 nm OBIS-CW, Coherent Optics) were aligned, expanded, and focused into the back focal plane of the objective (Nikon CFI 100 × 1.49 NA oil immersion) using a variety of dichroic mirrors, beam expanders, and lenses. Laser intensity modulation was controlled digitally by a computer via USB. A quad band dichoric mirror (ZT405/488/561/640rpc; Chroma) was used to separate fluorescence emission from excitation light. Fluorescence emission was further split into the far red and green signal using a dichroic longpass beamsplitter (FF652-Di01; Semrock) and band pass filters FF731/137 (Semrock) for the far-red channel and ET525/50 (Chroma) for the green channel. Programmable shutter sequences were inputted into an NI-DAQ board to synchronize laser output with camera frame duration. The HAL4000 software (Zhuang lab github https://github.com/ZhuangLab/storm-control) was used for intensity modulation, programmable shutter sequence input, camera settings, and image acquisition.

For imaging, a 10-frame shutter sequence at 20 Hz was employed to obtain conventional fluorescence, bright-field LED, and single molecule localization images. The first frame was the conventional GFP image obtained by exciting the sample with a 488 nm laser at 1.75 mW (power density ∼100 W/cm^2^). During the second frame a bright field LED image was recorded to observe cell morphology and health while a variable 405 nm intensity between 1–251 μW (power density of ∼ 0.06–15 W/cm^2^) was applied to the cell to photoactivate the HaloTag bound PA-JF646 dye. During the following 8 PALM imaging frames, single molecule signals were recorded by exciting cells using a 640 nm laser at 17.5 mW (∼1 kW/cm^2^). This shutter sequence was repeated for 15,000–30,000 frames and stopped when an aberrant cell morphology indicated declining cell health. Cells never changed their morphology for 15,000 frames and no more than 10,000 frames were used for the analysis to exclude potential phototoxic effects prior to changes in cell morphology. A bead calibration was employed to accurately map single molecule localizations identified in the 640 nm channel to the 488 nm channel to account for spherical aberrations created by the emission path (50,51).

For mEos2-NLS experiments, a similar imaging protocol was employed except a 561 nm laser was used for excitation instead of a 640 nm laser. In the emission path, fluorescence emission between the red and green channels were separated by a dichroic longpass beamsplitter (T562lpxr BS; Chroma) along with bandpass filters: ET525/50 (Chroma) for the green channel and ET595/50 (Chroma) for the red channel. Representative raw data of all experiments can be seen in supplemental videos 1–6)

### Single molecule localization, single molecule tracking, mean squared displacement and diffusion coefficient estimation

The Insight3 software from Xiaowei Zhuang's group was used to identify single molecule localizations and to fit them with 2D Gaussians with the following parameters: 7 × 7 pixel ROI, widths between 250–700 nm, and minimum 100 photons. The x- and y coordinates of the localizations, along with the intensity, width, background, frame number and other parameters were obtained for each identified single molecule. For the 60 Hz MCP-HaloTag experiments and 20 Hz mEos2-NLS experiments, localizations were identified with intensity values above 30 and 50 photons respectively with all other parameters staying similar to the parameters above. For each imaging condition, a minimum of 3 different cells taken at different days was used for analysis. Localizations were obtained as a text file from the Insight3 software and all other analysis codes were written in MATLAB 2018b.

Localizations that were within 0.48 μm of each other in consecutive frames were linked together to a trace. A low number of molecules was activated in each frame to prevent false linking (Supplementary Figure S1A, S1B). Traces with a minimum of 4 localizations in consecutive frames were used for cross-correlation and single trace fitting analysis. No dark frame was allowed between localizations. Displacements for each time interval in a single trace were averaged to obtain a time averaged mean squared displacement (TAMSD) vs time plot. Each TAMSD vs time step plot was fitted to the 2D diffusion equation <r^2^> = 4DΔt+ 2σ^2^, where D is the diffusion coefficient, Δt is the time step, r^2^ is the TAMSD, and σ is the localization precision.

The diffusion coefficient distributions of MCP-HaloTag with and without telomere gRNA (Supplementary Figure S2A) taken at 20 Hz were statistically similar to diffusion coefficient distributions of MCP-HaloTag with and without telomere gRNA taken at 60 Hz (Supplementary Figure S2B). The statistical similarity of the distributions was quantitatively assessed using the Kologmorov-Smirnov Test (KS test) (*P* = 0.72 no gRNA, *P* = 0.65 telomere gRNA). However, the diffusion coefficient distributions were shifted towards lower values in the data taken at 20 Hz compared to 60 Hz. Similar findings were also observed when comparing diffusion coefficient distributions of dCas9 with and without globally repetitive target gRNAs and telomere binding proteins taken at faster frame rates (50–500 Hz) (27,32,33,42,52). This indicates that diffusion coefficients of MCP-HaloTag taken at 20 Hz frame rates can identify molecules in their different mobility states but misses fast diffusing unbound molecules that are unwanted. While faster PALM imaging speeds would in principle reduce motion-blurring, there are some practical limitations. One the one hand, higher frame rates and higher excitation power would reduce the length of single molecule traces and thus lower the precision for characterizing their motion. In the theoretical extreme case where a fluorophore bleaches within one frame, single molecule tracking would not be possible. On the other hand, most PALM compatible fluorophores can reversibly transition to dark states that are unaffected by the excitation power. To avoid spatio-temporal overlap of multiple fluorophores, the 405 nm photoactivation rate is limited, which in turn limits possible imaging speeds. The used 20 Hz frame rate has therefore been in our experience a good compromise between imaging speed, good localization precision in each frame and obtained single molecule trace lengths (Supplementary Figure S3A and B.). Localization precision was calculated using the Thompson resolution formula (53).

### GFP cluster identification, tracking and diffusion coefficient estimation

Telomere GFP clusters were also identified using Insight3 software. GFP localizations were obtained by fitting GFP clusters with 2D Gaussians and the following parameters: 11 × 11 pixel ROI, widths between 250–7000 nm and a minimum of 200 photons. The mean GFP localization width of telomeres across all analyzed cells was 376 +/− 221 nm and the maximum GFP localization width was 4181 nm. X- and y- coordinates of the GFP localization, along with the intensity, width, background, frame number and other parameters were again obtained for each identified localization.

For telomere cluster tracking experiments, GFP localizations that were within 0.68 μm of each other in consecutive conventional imaging frames (every 10 frames) were linked together to form a trace. Traces with a minimum of 5 localizations were used for downstream analysis. The widths of consecutive localizations in a trace had to be within 200 nm of each other in order for the trace to be included in downstream analysis. An axial bead calibration showed that PSFs width deviations of 200 nm corresponded to axial deviations of 450 nm which is similar to the lateral trace linking threshold (54). A linear interpolation between the x- and y- coordinates of GFP localizations in consecutive conventional image frames (frame 1 and frame 10) was employed to obtain GFP coordinates during the frames that contained single molecule localizations. In order to estimate interpolation errors, GFP telomere clusters were conventionally imaged for 200 seconds at 20 Hz. The telomere positions between frame 1 and frame 10 were again interpolated and compared to the actual position of the telomere in frames 2–9. The median interpolation error is 45 +/− 10 nm and the mean interpolation error is constant up to 20 frames (Supplementary Figure S4A and S4B).

Mean squared displacements and diffusion coefficients were calculated using the same procedure described in the single molecule identification and tracking section.

### Spatio-temporal cross-correlation between single molecule localizations and GFP cluster coordinates and Motion Correction PALM

In order to superimpose the single molecule and GFP localizations for cross correlation analysis, single molecule coordinates were transformed from the 640 nm (top) to the 488 nm (bottom) camera channel. A bead calibration was performed to account for spherical aberrations between the top and bottom camera channel. Multi-wavelength excitable beads (TetraSpeck microspheres, Invitrogen T7279) were placed on a slide and excited at both 488 nm and 640 nm wavelengths. The positions of the beads in both the top and bottom channel were fitted to a 3^rd^ order polynomial function to extract the coordinate transformation matrix between the two channels (50,51). Coordinates from at least N = 5 images each with at least 10 sparsely distributed beads over the entire field of view were used to construct the transformation matrix. The bead calibration measurements were performed every day before imaging and achieved a 15 nm precise registration (50,51).

Once single molecule coordinates were transformed, a distance matrix between the interpolated GFP localizations and all single molecule localizations in one frame were calculated. The radius of the last GFP localization prior to interpolation was used as the radius of the interpolated GFP cluster coordinates. Single molecule localizations whose distance to a GFP cluster was smaller than the radius of the cluster plus the localization precision of the cluster and the single molecule localizations were identified as co-localized with telomeres and isolated for further downstream analysis.

Only single molecule localizations that were part of trajectories with at least 4 localizations in consecutive frames were used for further analysis. Single molecule traces where all localizations resided inside a cluster were identified as bound traces. Single molecule traces where some but not all localizations resided inside a cluster were identified as partially bound traces. Single molecule traces where none of the localizations resided inside a cluster were identified as unbound traces. To correct for motion of telomeres in PALM images, the coordinates of GFP localizations were subtracted from coordinates of the single molecule localization. Once all the single molecule localizations were motion corrected, a convex hull was applied to the motion corrected localizations in a cluster to find the cluster boundary. This boundary was used to calculate the area. The extension of a cluster was determined to be the furthest distance between two motion corrected localizations that resided within one telomere. Due to the constant photoactivation rate, the number of localizations in each cluster was normalized by duration of that cluster in the field of view (Supplementary Figure S5A, S5B). The normalized number of localizations was used to calculate the localization density.

### Mobility state clustering

The single molecule analysis using unsupervised Gibbs sampling (SMAUG, written by the Julie Biteen group) was employed to cluster single molecule trace steps into distinct mobility states (55). For each trace category, all traces, bound traces, partially bound traces, and unbound traces were inputted into the program for mobility state analysis. Each condition was analyzed through SMAUG for 50000 and 100000 iterations 3 separate times to ensure that the algorithm converged to the same number of states and similar weight fractions (Supplementary Figure S6). The spot-on analysis was used to fit displacement distributions and to estimate diffusion coefficient values of identified mobility state populations. Spot-on accurately estimates the diffusion coefficient of traces that exhibit Brownian or sub-diffusive motion. The number of states (two) used for spot on analysis was chosen from the analysis of mobility state convergence plots from SMAUG (Supplementary Figure S6). To compare the diffusion coefficients from the spot-on analysis to the ones obtained by linear MSD fitting of single molecule traces, a Gaussian mixture model was fitted to the log diffusion coefficient distributions obtained from linear MSD fitting (Supplementary Figure S7, see also Figure 3A). The Bayesian information criterion was used to assess goodness of fit.

## RESULTS AND DISCUSSION

### Motion of telomeres prevents high-resolution PALM measurements in living cells

**Figure 1.**
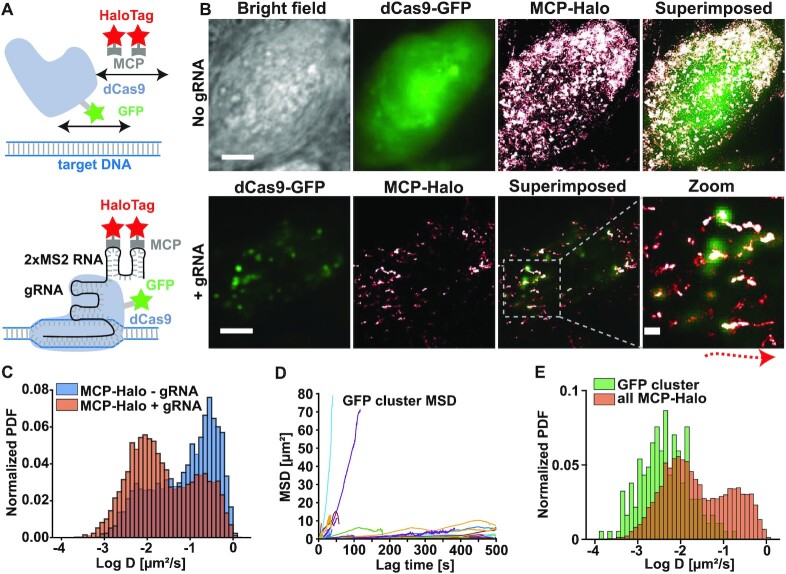
Motion of telomeres prevents high-resolution PALM measurements in living cells. **(A)** Fluorescent labeling strategy of repetitive telomere sequences involving GFP-tagged dCas9 for conventional fluorescence imaging and MCP-HaloTag proteins that bind to the two MS2 sites of the guide RNA and form a complex with dCas9 for PALM imaging (lower). **(B)** Upper: In the absence of gRNA the conventional fluorescence signal of dCas9-GFP (green) as well as the PALM localizations of MCP-HaloTag (red/white) are distributed throughout the nucleus of a cell. Lower: In the presence of gRNA dCas9-GFP and MCP-HaloTag bind to and form a complex at the telomere sites as evident by the presence of bright puncta in the GFP and PALM images. The magnified superposition shows clear telomere cluster in the GFP image while the PALM localizations are smeared out at various degrees along the paths of the telomeres during the long PALM data acquisition time. Since the conventional GFP images are averaged over 50 frames and since the displayed localizations are from 10000 frames, only partial co-localization is visible (scale bar: 5 μm; scale bar zoom: 800 nm; arrow indicates motion of telomere). **(C)** The diffusion coefficient distribution from single MCP-HaloTag proteins significantly overlaps in cells with and without telomere gRNA (7281 traces with telomere gRNA and n = 5749 traces without gRNA from N = 5 cells). **(D)** The mean squared displacement of telomere GFP traces exhibits a wide variability with some immobile telomeres and others that are actively transported (n = 81 traces from N = 5 cells). **(E)** The diffusion coefficient distributions of GFP telomere traces and single MCP-HaloTag traces show some degree of overlap (N = 5 cells).

In order to demonstrate the challenges of conventional PALM imaging to assign localizations to a locus and to extract structural information in living cells, we targeted the highly repetitive telomere sequence that has been previously characterized with conventional fluorescence and PALM imaging (13–15,29,31). We transiently expressed a GFP-tagged dCas9 for conventional fluorescence imaging in addition to the MCP-HaloTag protein, which was conjugated to the PALM compatible PA-JF646 dye, binds to the gRNA MS2 sites, and forms a complex with dCas9-GFP (Figure 1A). In the absence of gRNA, both the conventional fluorescence signal of dCas9-GFP and the single molecule localization from MCP-HaloTag were distributed throughout the nucleus (Figure 1B). This result is expected since both fluorescent reporters cannot bind to the telomere regions in the absence of gRNA. Importantly, the single molecule localizations exhibited some degree of clustering, which highlights the difficulty to identify which localizations are bound to a locus just based on their density. When the gRNA was expressed, the conventional fluorescence signal showed pronounced puncta indicating dense binding of dCas9-GFP to telomeres as in previous studies (13,15,16,31). The single molecule localizations in the PALM images also formed dense regions that partially overlapped with the conventional fluorescence puncta. However, the single molecule localizations were smeared out to various lengths along the movement of the telomeres during the long PALM data acquisition time. This result demonstrates that high-resolution structural information cannot be obtained via PALM imaging in live cells and that the motion of loci needs to be corrected.

With the same PALM data, we performed single molecule tracking of MCP-HaloTag by linking localizations that appeared within a distance threshold in consecutive frames (see Materials and Methods). From the resulting single molecule traces, the time-averaged mean squared displacements (MSDs) were calculated to obtain a first-order estimation of diffusion coefficients though linear MSD fitting (see also Materials and Methods). Telomeres, as part of the chromosome polymer, are generally expected to exhibit non-Brownian anomalous sub-diffusion with anomalous exponents alpha < 1 (56,57). However, due to the difficulties of fitting the anomalous exponent from short and noisy trajectories (58), we assume Brownian motion in our trajectory analysis for simplicity in this paper as in many other studies (27,31,33,42,52,59–61) and note that this assumption may affect downstream analyses. Thus, while the obtained absolute values of diffusion coefficients are not accurate since they depend on the frame rate (e.g. comparison between 20Hz and 60 Hz in Supplementary Figure S2A and S2B) and the used analysis model, they provides a good approximation to discriminate fast from slow or sub-divisively moving molecules and to make relative comparisons.

In the presence of gRNA, the distribution of MCP-HaloTag proteins exhibited a pronounced peak at large diffusion coefficients around 0.21 +/− 0.01 μm^2^/s, indicating fast diffusion of searching or unbound probes. In addition, the diffusion coefficient distribution contained a peak at small diffusion coefficients around 0.01 +/− 0.003 μm^2^/s, which could be interpreted as the mobility of bound probes (Figure 1C). The distribution of diffusion coefficients in the absence of gRNA also showed a peak at large diffusion coefficients (0.28 +/− 0.03 μm^2^/s) but in addition contained a significant fraction of small diffusion coefficients. Importantly, these small diffusion coefficients overlapped with the slow fraction of bound MCP proteins in the presence of gRNA. The overall overlap between both distributions of 71.5% demonstrates that single molecule traces from the dCas9/MCP complex cannot be classified as bound just by their diffusion coefficient. This issue is further compounded by the increased uncertainty of diffusion coefficients obtained by fitting short traces due to increased effect of the localization error. To validate linear MSD fitting for estimating diffusion coefficients, we analyzed the single molecule traces with the spot-on analysis (62), which can accurately characterize the mobility of single molecule trajectories that exhibit Brownian or sub-diffusive motion. Spot-on identified two mobility populations with two diffusion coefficients that closely resembled and thus validated the two peaks of the diffusion coefficient distribution obtained by linear MSD fitting (Supplementary Figure S7). It is important to note that the diffusion coefficient distribution of mEos2 with a nuclear retention signal showed the same slow diffusing fraction as MCP in the absence of gRNA. (Supplementary Figure S2C). The Kolmogorov Smirnov test performed at a 0.05 significance level showed that the diffusion coefficient distributions are not statistically different (*P* = 0.79). Therefore, the slow fraction of MCP proteins in the absence of gRNA is not caused by non-specific protein-RNA interactions but is likely due to the inhomogeneous and highly crowed environment of the nucleus that can have a wide range of apparent viscosities (27,31–33,63,64).

In order to quantify the mobility of telomeres and to determine the extent of motion blurring in PALM images, we tracked the conventional fluorescence signal that was recorded every 10th frame of the PALM imaging sequence. The resulting mean squared displacement vs. time traces exhibited a very wide range of slopes. Some telomeres were almost immobile, while others were actively transported over a distance of up to 10 μm (Figure 1D). This heterogeneity of telomere mobilities highlights the need to correct PALM localizations of each individual telomere for motion in order to obtain time-averaged structural information. To obtain a first order approximation of the telomere motion that can be compared to the one from single molecule traces, we again performed linear MSD fitting as previous studies (31,33,42,59–61). While the obtained absolute values of diffusion coefficients are not accurate due to the different types of motion, they do allow a relative discrimination of fast and slowly moving telomeres. The peaks of the two distributions identified by a Gaussian mixture model were again close to the values identified by spot-on analysis, which estimates the mobility of traces exhibiting Brownian or sub-diffusive motion (Supplementary Figure S7). Furthermore, the diffusion coefficient distribution of telomere traces exhibited partial overlap with the bound fraction of single MCP traces (Figure 1E). However, single molecule traces were generally faster, which can be caused by a relative motion with respect to the center of mass of telomeres and the additional underlying motion of the telomeres themselves. The heterogeneity of the diffusion coefficients of individual telomeres therefore causes errors in quantifying the mobility of bound MCP molecules and in identifying bound MCP molecules based on a mobility threshold.

In summary, the chromatin mobility and the overlapping diffusion coefficient distributions of bound and unbound dCas9/MCP complexes result in errors for identifying bound single molecules based on their mobility and for quantifying their mobility. Furthermore, the wide range of telomere mobilities results in significant spreading of their bound single molecule localizations, preventing the extraction of structural parameters. These results demonstrate the need to employ a correlative conventional fluorescence and PALM imaging approach to dynamically identify these bound molecules throughout the long PALM data acquisition time and to correct for motion.

### Correlative conventional fluorescence and PALM imaging identifies bound molecules and corrects for chromatin motion

**Figure 2.**
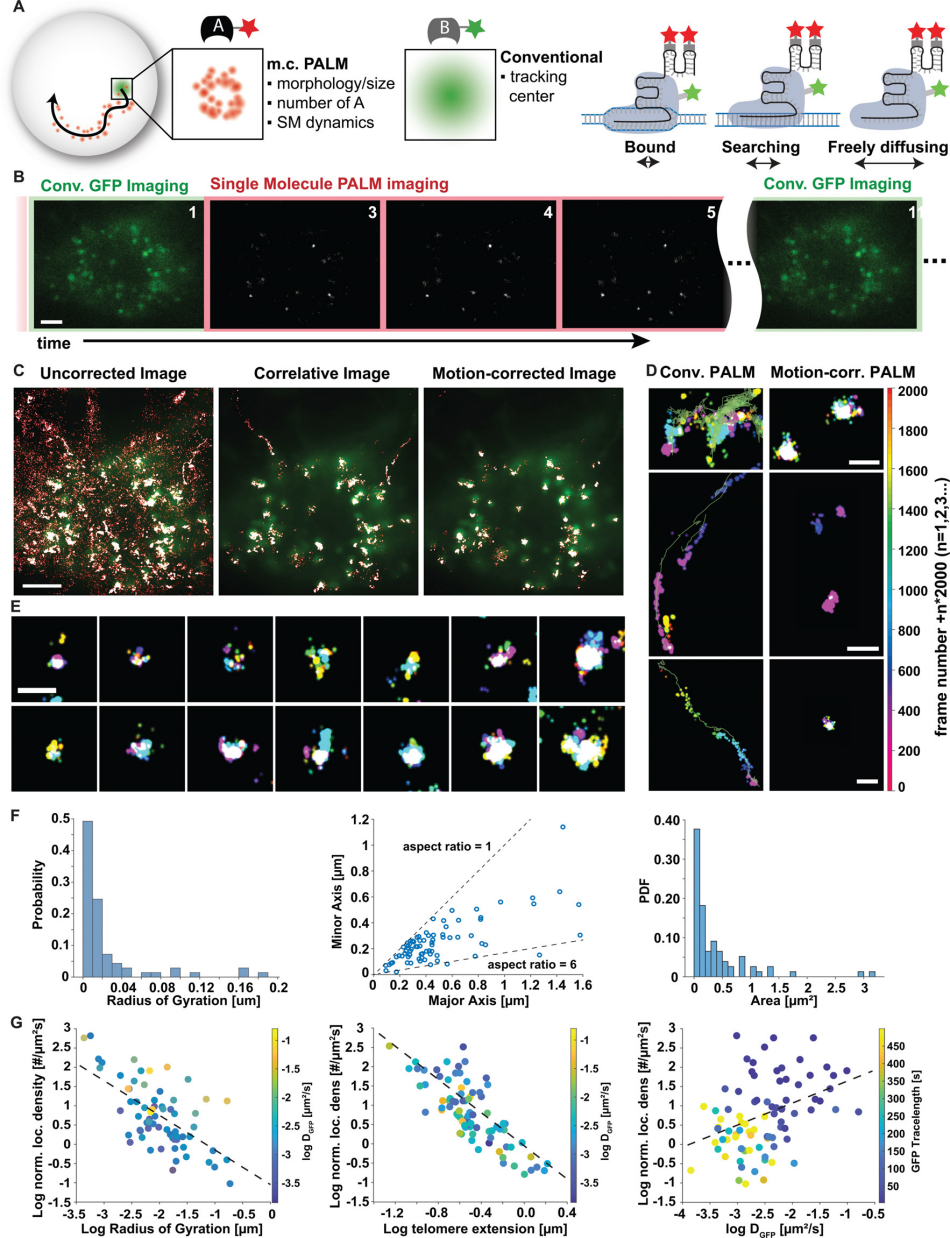
Correlative conventional fluorescence and PALM imaging identifies bound molecules and super-resolves moving telomeres. **(A)** Schematics of motion correction via correlative conventional and PALM imaging. Left: Single molecule localizations are smeared out along the trajectory of a locus, which is simultaneously tracked in the GFP channel. By subtracting the trajectory from PALM localizations the moving locus can be super-resolved but is averaged over time and potentially over radial movements. Right: In addition, correlative imaging can better assign bound localizations at any instance in time by the proximity to the conventional GFP signal and reduces background from searching and freely diffusing probes. **(B)** In a correlative conventional and PALM imaging sequence, GFP is excited and imaged in one frame followed by one frame of 405 nm photoactivation and brighfield imaging (not shown) and 8 frames of PALM imaging, in which bright single molecule fluorescent signals become visible. Scale bar represents 5 μm. **(C)** Left: Superposition of a GFP image and conventional PALM image that includes a majority of freely diffusing and searching fluorescent probes. Middle: The correlative conventional and PALM image only depicts PALM localizations that appear in proximity to a GFP cluster at any instance in time and suppresses background from freely diffusing and searching probes. Right: The motion-corrected PALM image super-resolves each moving telomere, which co-localizes with its GFP signal. More telomeres are visible in the GFP channel since they are detected over a wider z-range compared to PALM. Scale bar represents 5 μm **(D)** Magnifications of correlative PALM and motion-corrected PALM images show localizations of telomeres smeared out to various degrees (left) and super-resolved telomeres (right). Localizations are temporally color-coded by their appearance in a 2000 frame interval. Telomere trajectory is represented by the green line. Scale bars: 1 μm. **(E)** Individual examples of motion-corrected telomeres show broad variability in size, asymmetry and density. Scale bars: 1 μm. **(F)** Quantification of structural telomere parameters such as their radius of gyration (left), minor vs. major axis by elliptical fitting (middle) and area (right). **(G)** The localization density of individual telomeres shows a negative correlation with the radius of gyration (correlation coefficient −0.7, left) and the maximum extension of telomeres (correlation coefficient = - 0.8). The color of each telomere indicates no clear dependence on the GFP diffusion coefficient. The localization density of telomeres shows a slight correlation (correlation coefficient = 0.4, right) with their diffusion coefficient. The color represents the temporal trace length of the GFP telomere signal and indicates that slower telomeres can be imaged for a longer time. All data taken is from n = 81 clusters across N = 5 cells.

In order to correct for telomere motion during the long PALM data acquisition time and to reliably identify bound single molecules, we developed a correlative conventional and PALM imaging approach. In this approach telomeres are labeled with a conventional fluorophore such as GFP to continuously track their trajectory throughout the entire data acquisition time. In addition, telomeres are labelled with a spectrally distinct PALM dye (PA-JF646 bound to MCP HaloTag) to localize sparse single molecule signals, which get smeared out along the trajectory of a telomere. By subtracting the trajectory of the telomere from the single molecule coordinates, the motion can be corrected, and super-resolution images can be recovered and further quantified (Schematics in Figure 2A).

To minimize bleaching of dCas9-GFP, we employed a repetitive 10 frame shutter sequence, in which GFP is excited in one frame followed by photoactivation in the next frame and 646 nm excitation in the remaining 8 frames. As seen in Figure 2B, telomeres were detected and localized as continuously fluorescing puncta during the GFP excitation frames, whereas sparse and bright single molecule fluorescence bursts were detected during the remaining PALM frames. These bursts were then localized by gaussian fitting as in conventional PALM data analysis and rendered as gaussians with a width equal to the localization precision. The resulting PALM images exhibited single molecule localizations throughout the nucleus with some clustered regions and dense tracks along the motion of telomeres (Figure 2C, left). However, the effect of motion blurring and the background localizations of unbound molecules prevented the extraction of structural parameters and the reliable identification of molecules bound to telomeres. To assign single molecule localizations to a telomere, the GFP localizations were linked to trajectories of individual telomeres and linearly interpolated during the PALM frames (see Materials and Methods). While the localization precision of single molecules was estimated with two different metrics to be about 20 nm (Supplementary Figure S3), it is important to note that the estimated interpolation error of the GFP signal with a median of 45 +/− 10 nm over 10 frames (Supplementary Figure S4) dominates the uncertainty in resolution or structural quantification. Single molecule localizations were then assigned to the nearest telomere GFP localization if they were separated by less than the radius of the GFP cluster. In addition, the single molecule localizations were linked to traces to further exclude MCP molecules in their freely diffusing or search state on the DNA. If a molecule entered a telomere but remained bound for at least four frames, it was included. If a molecule entered and exited a telomere, it was excluded and interpreted as a searching or freely diffusing molecule (see Materials and Methods). This assignment of traces is based on previous studies showing that the residence time of bound dCas9, the stability of gRNA and the residence time of MCP bound to the gRNA is on the order of minutes to hours (21,27,32,65,66). It is therefore very unlikely to observe binding- and unbinding events during a short single molecule trajectory. On the other hand, the dCas9 residence time during scanning has been measured to be 20–30 milliseconds in prokaryotic genomes and between 20–100 ms in eukaryotic genomes (28,30,32). Based on these experimentally determined upper and lower time limits and the uncertainty for calculating the mobility for shorter traces, a 4 frame cutoff was chosen as a compromise to accurately determine the mobility state of a protein and to not falsely assign a scanning protein to be bound to the locus.

In the resulting PALM image depicting only localizations that were bound to a telomere, most background localizations are suppressed, and motion-blurred telomeres become clearly visible (Figure 2C, middle). After subtracting the GFP-trajectories of individual telomeres from their single molecule localizations, motion blurring is corrected, and a time-averaged super-resolution image of each telomere is obtained (Figure 2C, right). Importantly, motion is corrected with respect to the center of mass of each telomere at each point in time since all fluorophores of a telomere are excited simultaneously in the conventional fluorescence image. Therefore, motion correction accounts for potential nanoscale re-arrangements that are quantified in a later section. In contrast, averaging the single-molecule frames even up to 100 frames is not sufficient to accurately determine the center of mass of telomeres due to the sparse photoactivation and would in addition result in lower temporal and spatial resolution (Supplementary Figure S8). The individual examples shown in Figure 2D and Figure 2E highlight how this method can correct for the significant motion of a telomere and recovers the time-averaged super-resolved structure of a locus.

The motion-corrected super resolution image of each telomere provides quantitative structural information such as the degree of chromatin compaction and condensation. For instance, the radius of gyration, area, and minor vs. major axis quantify the size of each motion-corrected telomere and its asymmetry averaged over the time of data acquisition and potentially averaged over rotational degrees of motion (Figure 2F). The time-averaged size measurements of telomeres obtained with our method are consistent with previous measurements in chemically fixed cells (13,29,31,67–69). However, it is important to note that differences in telomere size can be due to differences in telomere lengths in different cell types, cell states, different chromatin states, or expansion or compaction of loci during live cell imaging (13,31,67–70). This information can then be related to other quantities such as the number of localizations bound to a locus to obtain insights into the density of bound probes (Figure 2G). Importantly, since the photoactivation rate for MCP-HaloTag localizations was held constant during imaging, the number of localizations has been normalized by the observation time of telomeres (Supplementary Figure S5A and B). Localization densities of telomere clusters of varying temporal trace lengths can therefore still be compared without bias. However, telomeres with a large diffusion coefficient were observed a shorter time than slow telomeres since faster telomeres are more likely to move out of focus during the data acquisition time (Figure 2G). We observed an overall trend that smaller, less extended telomeres had a higher localization density whereas larger and more extended telomeres exhibited a lower localization density (Figure 2G left: correlation coefficient = −0.7; middle: correlation coefficient = −0.8). Since the maximum extension of telomeres depends to some degree on their area this observation can be explained by less extended telomeres being more compact and exhibiting a higher density of bound fluorophores and by more extended telomeres having a lower fluorophore density (Supplementary Figure S5C). It is also important to note that the measured telomere area is potentially radially averaged. However, since telomeres with a certain maximum extension can deviate in their area by up to an order of magnitude (Supplementary Figure S5C), and since aspect ratios of over 5 are observed (Figure 2F) some asymmetry is still conserved.

While localization density measurements of chromatin have been performed in fixed cells in the past and used as a measure of chromatin condensation (29,31,60,67), the power of motion-correction PALM is to perform such measurements in living cells and to simultaneously obtain dynamic information about the motion of individual telomeres and of individual bound fluorescent probes. We therefore related the localization density of each telomere to their mobility determined from their GFP signal. The mobility of telomeres did not show a tight correlation with their localization density (correlation coefficient = 0.39), however, slowly moving telomeres generally had a lower localization density while faster telomeres had a higher localization density (Figure 2G, right). This observation can be explained by the ability of dense telomeres that are generally also less extended to move more freely while less dense and larger telomeres might be more restricted in their overall movement. This is consistent with previous studies comparing indirect telomere size measurements and mobility (13). Variations in telomere mobility across similar sizes and densities could be explained by local changes in the chromatin environment (13).

These results demonstrate that motion-correction PALM is able to provide quantitative time-averaged structural insights into the compaction and condensation of chromatin and to relate this structural information to the dynamics of chromatin in living cells.

### Reliable identification of single molecule traces that are bound to moving loci

Correlative conventional and PALM imaging not only yields time-averaged structural information of chromatin as shown above, but can also be applied to study how single dCas9/MCP complexes bound to DNA move relative to the larger chromatin domain they reside in. Recent studies highlight the importance of characterizing the degree of correlation between small- and large scale chromatin motion and its relation to gene regulation and phase separation (6–10). A prerequisite for measuring relative DNA mobility is the ability to reliably identify dCas9/MCP complexes that are bound to a locus and to separate them from searching and freely diffusing ones. Since the diffusion coefficient distributions of these mobility states overlap (Figure 1C), it is not possible to identify bound dCas9/MCP complexes just based on their mobility. Here, we demonstrate that correlative conventional and PALM imaging reliably identifies traces of single dCas9/MCP complexes bound to chromatin to better characterize their mobility without imposing any threshold on diffusion coefficients.

**Figure 3.**
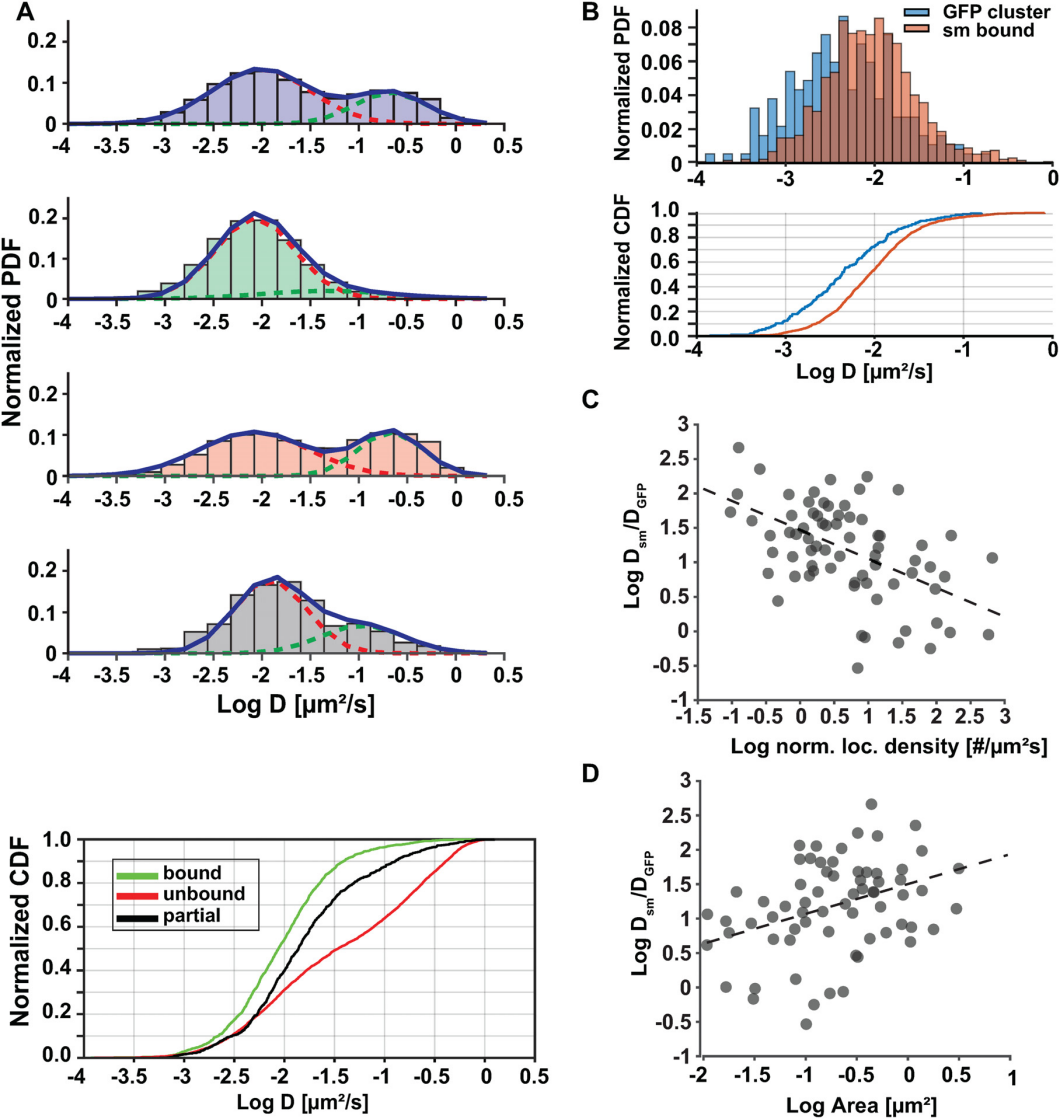
Accurate identification of bound dCas9 molecules reveals their telomere-specific relative mobilities. **(A)** Upper: Diffusion coefficient histograms of all dCas9-MCP complexes and of molecules identified with correlative conventional and PALM imaging to be bound (n = 1490 traces), unbound (n = 5077 traces) and partially bound (n = 714 traces) to telomeres. Lower: Cumulative diffusion coefficient histograms of the same classes of molecules. There is significant overlap of all distributions. **(B)** Upper: Diffusion coefficient histogram of telomeres (GFP) and dCas9-MCP complexes identified to be bound. There is significant overlap, but generally single molecules move faster than the telomeres they reside in. Lower: Cumulative diffusion coefficient histogram of the same classes. **(C)** The ratio of average dCas9-MCP diffusion coefficients and the telomere they reside in shows a negative correlation (correlation coefficient = - 0.52) with the normalized localization density of the telomeres. **(D)** The ratio of average dCas9-MCP diffusion coefficients show a positive correlation (correlation coefficient = 0.48) with the area of the telomeres they reside in. All data was collected from N = 5 cells and there were no statistically significant differences in the group comparisons between cells (multi-way ANOVA *P* = 0.35).

Using correlative conventional and PALM imaging, dCas9/MCP traces were classified as bound, unbound and partially bound based on their proximity to a conventional fluorescence signal from a telomere at each point in time as described in the previous section (see also Materials and Methods). The diffusion coefficient distribution of bound trace were unimodal with one peak at 0.008 +/− 0.002 μm^2^/s while the unbound and partially bound traces exhibited two peaks (unbound: 0.008 +/− 0.002 μm^2^/s and 0.210 +/− 0.005 μm^2^/s; partially bound: 0.01 +/− 0.005 μm^2^/s and 0.11 +/− 0.007 μm^2^/s) as determined with a two state Gaussian fit (Figure 3A). The diffusion coefficient distribution of unbound traces had significant overlap with the one of bound traces, highlighting the inability to classify bound dCas9/MCP complexes just based on their mobility. For instance, the normalized probability density distribution of bound and unbound traces had a 62% overlap (Supplementary Figure S9A and S9B). Correlative conventional and PALM, however, can reliably separate slowly moving unbound traces from bound dCas9/MCP complexes. These results demonstrate that an accurate classification of dCas9/MCP complexes can be used to study their dynamics while freely diffusing, searching on DNA, and while being bound to a target locus.

The advantages of accurately classifying bound traces also manifest themselves when comparing the motion of individual dCas9/MCP complexes to the motion of the entire telomere they reside in. The diffusion coefficient distributions of all dCas9/MCP traces only exhibit a partial 68.5% overlap with the ones of the telomere cluster traces (Figure 1E). However, the traces classified with correlative imaging to be bound show a larger overlap of 75% and some degree of correlated motion (Figure 3C). Both diffusion coefficient distributions resemble a Gaussian distribution on a log scale and indicate sub-diffusive mobility, which is a hallmark of chromatin mobility. While bound dCas9/MCP traces and entire telomeres exhibit sub-diffusive mobility, the median of the diffusion coefficient distribution of the bound traces (0.007 +/− 0.003 μm^2^/s) is larger than the one of telomere cluster traces (0.002 +/− 0.0005 μm^2^/s). We hypothesize that this difference is because DNA at the nucleosomal level tends to move faster than its larger domain counterpart. In contrast, the skewness in the diffusion coefficient distribution of partially bound traces compared to bound dCas9/MCP and telomere traces indicates that the mobility of these molecules is not sub-diffusive. Therefore, partially bound molecules are not likely bound to DNA but likely diffusing through a telomere cluster or searching for a binding site. This interpretation is further supported by previously reported residence times of dCas9 on DNA on the order of minutes to hours, which is much longer than the bleaching time of fluorescent probes used for localization microscopy (27). In addition, nucleosomes have been shown to kick off scanning dCas9 molecules and shorten search state residence times to milliseconds in eukaryotic cells (27). Because of these two facts, it is very unlikely to observe a dCas9/MCP complex bind to and leave its target site during the length of a single molecule trace.

We next evaluated how fast on average single dCas9/MCP complexes move relative to the larger telomere domain they reside in. The average diffusion coefficient of all bound traces in a telomere cluster was first calculated by fitting the averaged MSDs to the 2D diffusion equation and then divided by the diffusion coefficient of the telomere. In almost all cases, single dCas9/MCP complexes moved faster than their larger telomere domain they resided in (Figure 3C and D). In a few cases the single molecule traces were coincidentally obtained during sections where the mobility of the cluster was faster than the average mobility of the telomere cluster. Telomeres with a low localization density exhibited the fastest relative mobility of single dCas9/MCP complexes and the largest variance in their relative mobilities. dCas9/MCP complexes in dense telomeres had a low or no relative mobility and a narrower range of diffusion coefficient ratios (Figure 3C and Supplementary Figure S9E). These results indicate that less dense telomeres undergo a high degree of dynamic chromatin rearrangement while dense telomeres exhibit a more static chromatin state potentially due to tighter interactions. When comparing the relative diffusion coefficient with the area of telomeres, a slight correlation is observed. Smaller clusters that are potentially denser showed the lowest relative mobility and variance in diffusion coefficients of single molecules whereas larger clusters showed the highest relative mobilities and variance in diffusion coefficients (Figure 3D and Supplementary Figure S9F). This result is consistent with the previous finding that denser telomeres are smaller (Figure 2E and Supplementary Figure S5C) and again indicates that larger, less compacted telomeres undergo more chromatin remodeling compared to small telomeres.

In summary, these results demonstrate that correlative conventional and PALM imaging can reveal how chromatin compaction and condensation affects the motion of small nuclear rearrangements within a larger chromatin domain. Overall, this data shows that dCas9/MCP complexes move significantly faster than the larger telomere domains they reside in. We show that more compact clusters with a higher localization density, exhibit a higher degree in correlation in single molecule mobility and less variance compared to less dense clusters. These findings are consistent with single nucleosome tracking measurements showing that more compact chromatin domains move coherently (71–73). Our findings also match existing nucleosome tracking data that identified chromatin density as an important regulator of instantaneous chromatin dynamics (6). Since the relative mobility of chromatin is a hallmark of protein DNA phase condensation and gene regulation, our approach may aide in characterizing the formation of nuclear phase condensates in future experiments with appropriate controls (6–10,12).

### Mobility state analysis reveals hidden state in bound traces

A general limitation to obtain accurate diffusion coefficients from short single molecule traces is the increased effect of the localization uncertainty (74,75). Only an apparent diffusion coefficient can be obtained and used as a relative mobility comparison to other traces. To obtain a more accurate diffusion coefficient estimate of an entire mobility population, traces or individual step-sizes are therefore often clustered into specific mobility populations and the average diffusion coefficient of the population is calculated (55,62). To demonstrate that correlative conventional and PALM imaging can reveal mobility states that are hidden when all traces of conventional PALM data are analyzed, we utilized the Bayesian cluster analysis SMAUG to determine mobility states of dCas9/MCP complexes (55). We used this method over Gaussian mixture models, hidden Markov models, and other vibrational Bayesian clustering techniques due to the ability of the SMAUG algorithm to analyze short traces without the requirement of a predetermined number of mobility states for fitting single molecule data (55). However, a disadvantage is that a Brownian model is used to approximate the motion of traces that may be non-Brownian.

**Figure 4.**
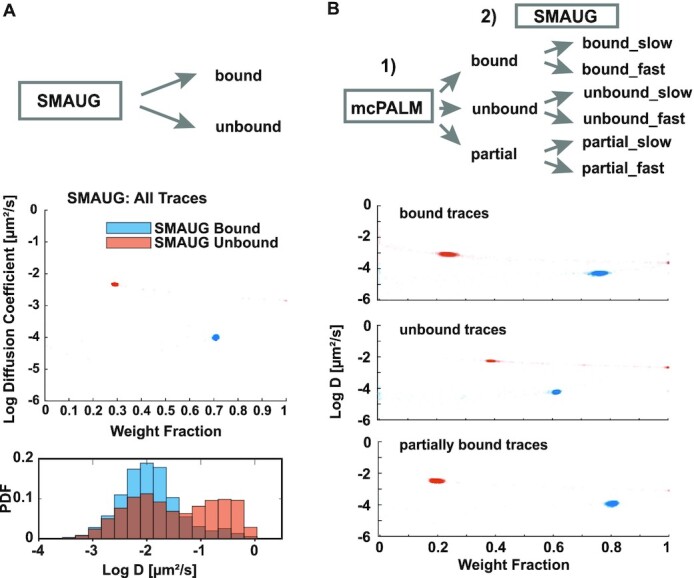
Correlative conventional and PALM imaging combined with SMAUG analysis reveals hidden mobility states. **(A)** Upper: When all dCas9-MCP traces are analyzed with SMAUG, a bound and unbound population is identified. Lower: calculating the diffusion coefficient distributions from the corresponding bound and unbound traces results in similar distributions as observed with correlative conventional and PALM imaging. **(B)** When correlative conventional and PALM imaging is performed first to separate bound, unbound and partially bound traces, SMAUG identifies in each class two mobility states that were not detected when all traces were analyzed. Data collected from N = 5 cells.

First, we analyzed the mobility states of all dCas9/MCP single molecule traces with SMAUG. The result in Figure 4A (upper left) shows the weight fraction of the mobility state classification and the apparent diffusion coefficient of each mobility state from the last 20,000 iterations of the SMAUG algorithm (see also Supplementary Figure S6). SMAUG identified a bound and faster unbound state with clear convergence. However, it is important to note that the absolute values of diffusion coefficients obtained by SMAUG do not match the ones obtained by mean squared displacement fitting due to its algorithm that takes into account localization uncertainties and only analyzes displacements of single molecules in consecutive frames. Therefore, only the relative classification of traces into different mobility states was used to then calculate the diffusion coefficient distribution based on MSD fitting (Figure 4A, lower). The traces classified by SMAUG to contain bound step sizes exhibit a similar distribution as the ones obtained from the correlative approach with a peak at 0.01 μm^2^/s (Figure 3A). Likewise, the traces classified as unbound exhibited a bimodal distribution with an additional peak at 0.5 μm^2^/s. This result is consistent with the control experiment in Figure 1C in the absence of gRNA, where a slow diffusing unbound population of dCas9/MCP was observed. These two peaks in the diffusion coefficient distribution are not visible in the overall assignment of mobility states by SMAUG (Figure 4A, upper) since its algorithm uses displacements in consecutive frames and assigns an averaged diffusion coefficient to the population. Therefore, a trace containing a step of the unbound population could have other shorter steps that result in a slow diffusion coefficient from its MSD.

We next applied SMAUG to the three classes of traces identified with correlative conventional and PALM imaging (bound, unbound and partially bound) to obtain a more refined characterization of mobility states in each class. When the algorithm was applied to the traces identified by motion correction to be bound (Figure 4B), SMAUG identified 2 mobility states. This result could be explained by the heterogeneity in telomere mobility itself and is consistent with other dCas9 and nucleosome imaging data (Figure 1D and E) (13,15,31,73). In both the unbound and partially bound trace populations, SMAUG again identified two mobility states. The fast mobility state identified in the bound population is distinct from the fast and slow mobility states identified by SMAUG in all traces, unbound, and partially bound traces. This shows that when paired with motion correction PALM, SMAUG can identify a fast and slow bound population that could not be identified otherwise due to the large overlap in mobilities between slowly moving unbound traces and the bound traces.

To complement the SMAUG analysis and to demonstrate the advantage of our correlative imaging approach for other downstream analysis, we also analyzed the bound, unbound and partially bound traces with spot-on (62). While spot-on can accurately characterize the mobility of single molecule trajectory populations that exhibit Brownian or sub-diffusive motion, it does require a user defined number of mobility states in contrast to SMAUG. Based on the SMAUG results we therefore imposed two mobility states for bound, unbound and partially bound traces and obtained diffusion coefficients and weight fraction for each state. The diffusion coefficient estimates from spot-on for each mobility state was similar to the peak values of a Gaussian mixture model fitted to the diffusion coefficient distribution from linear MSD fitting (Supplementary Figure S10). Likewise, the weights between the slow and fast mobility state identified by spot-on analysis also corresponded well to the weights of the Gaussian mixture model fitted to the diffusion coefficient distribution from linear MSD fitting. These results demonstrate that correlative conventional and PALM imaging when combined with other trace classification approaches enhances mobility state analysis by a more accurate identification of the bound population, which can also lead to a more accurate calculation of kinetic parameters. The advantages and disadvantages of existing methods for downstream single molecule mobility analysis highlight the usefulness to discriminate bound from unbound single molecules with correlative conventional and PALM imaging without the need of mobility thresholds. At the same time, the discrimination of bound and unbound traces with correlative imaging is advantageous for any existing downstream mobility analysis method and for any method developed in the future that may combine the advantages of existing techniques.

## CONCLUSIONS AND FUTURE OUTLOOK

The advent of PALM imaging presented new opportunities to study the structure and dynamics of chromatin in a new level of detail. Previous studies and technique developments yielded important insights into the nanoscopic chromatin structure in fixed cells and into its dynamics in living cells. However, available techniques cannot obtain both, structural and dynamic information in living cells due to the motion of chromatin during the long PALM data acquisition times. Here we developed and demonstrated a correlative conventional fluorescence and PALM imaging approach that tracks the motion of labelled loci in order to correct for their motion in the simultaneously acquired PALM images. We employed dCas9/MCP-based imaging of telomeres as a model system to demonstrate that motion-correction PALM allows for measuring both, time averaged super-resolved structural parameters as well as dynamics of chromatin organization such as various transport modes. This approach also yielded information into how individual dCas9/MCP complexes bound to DNA move relative to larger chromatin domains they reside in. Identifying bound dCas9/MCP probes using the correlative approach avoids the need of using a fixed mobility threshold and provides more accurate information about single molecule probes bound to chromatin. Probes identified as bound and unbound can then be further analyzed with downstream mobility analysis methods to obtain more refined results compared to when all single molecule traces are analyzed.

Our study lays the foundation for further refinement and optimization of the presented correlative conventional fluorescence and PALM imaging technique and its application to study a myriad of different chromatin loci. For instance, extending this technique to 3D to track loci for longer periods of time and to extend the lifetime of the conventional fluorescence signal would extend the imaging time of loci and result in an improved spatial and temporal resolution (15,31,60,76,77). In addition, orthogonal RNA binding proteins and/or CRISPR-Cas proteins could be applied to enable simultaneous multicolor imaging of different chromatin regions (14,19–21).

Of particular interest would be correlative conventional and PALM imaging of non-repetitive and locally repetitive chromatin loci due to the reliable discrimination of bound fluorophores from freely diffusing ones. Such experiments would require a conventional fluorescence signal for a sufficient period of time to accumulate enough single molecule localizations for the generation a motion corrected image. Many recently developed CRISPR based DNA labelling schemes already can track single chromatin loci on the order of minutes to hours (13–15,19,21,31,39,66,78) which would make them compatible with our method. These studies use a variety of labeling techniques to label DNA and typically require between 40–100 bound conventional fluorophores to generate a stable conventional image. The number of required photo-switchable fluorophores would need to be either similar to or less than the number of conventional fluorophores. From a labeling perspective, it would be easier to keep the number of fluorophores the same by appending a photo-switchable fluorophore to the conventional fluorophore.

Because dCas9 can only bind to accessible chromatin regions, characterizing the number of bound probes at a specific locus can shed light into the chromatin accessibility and show how it changes over time or in response to perturbations (65,79,80). Correlative conventional and PALM imaging therefore paves the way to study chromatin structure and dynamics of locally repetitive or non-repetitive loci in a new level of detail and to relate the obtained structural and dynamic parameters in living cells.

## DATA AVAILABILITY

Due to the large size of PALM data it cannot be deposited in public repositories but will be made available upon request. The code for motion correction PALM as well as data tables to create figures are available at https://doi.org/10.17605/OSF.IO/6N4EJ.

## Supplementary Material

gkac314_Supplemental_Files

## References

[1] Risca V.I. , Greenleaf W.J. Unraveling the 3D genome: genomics tools for multiscale exploration. Trends Genet. TIG. 2015; 31:357–372. 25887733 10.1016/j.tig.2015.03.010 PMC4490074

[2] Szabo Q. , Bantignies F., Cavalli G. Principles of genome folding into topologically associating domains. Sci. Adv. 2019; 5:eaaw1668. 30989119 10.1126/sciadv.aaw1668 PMC6457944

[3] Lakadamyali M. , Cosma M.P. Visualizing the genome in high resolution challenges our textbook understanding. Nat. Methods. 2020; 17:371–379. 32123395 10.1038/s41592-020-0758-3

[4] Klemm S.L. , Shipony Z., Greenleaf W.J. Chromatin accessibility and the regulatory epigenome. Nat. Rev. Genet. 2019; 20:207–220. 30675018 10.1038/s41576-018-0089-8

[5] Ramani V. , Deng X., Qiu R., Gunderson K.L., Steemers F.J., Disteche C.M., Noble W.S., Duan Z., Shendure J. Massively multiplex single-cell Hi-C. Nat. Methods. 2017; 14:263–266. 28135255 10.1038/nmeth.4155 PMC5330809

[6] Barth R. , Bystricky K., Shaban H.A. Coupling chromatin structure and dynamics by live super-resolution imaging. Sci. Adv. 2020; 6:eaaz2196. 32937447 10.1126/sciadv.aaz2196 PMC7458449

[7] Shaban H.A. , Barth R., Bystricky K. Formation of correlated chromatin domains at nanoscale dynamic resolution during transcription. Nucleic Acids Res. 2018; 46:e77. 29718294 10.1093/nar/gky269 PMC6061878

[8] Zhang Y. , Lee D.S.W., Meir Y., Brangwynne C.P., Wingreen N.S. Mechanical frustration of phase separation in the cell nucleus by chromatin. Phys. Rev. Lett. 2021; 126:258102. 34241518 10.1103/PhysRevLett.126.258102 PMC8604804

[9] Lee D.S.W. , Wingreen N.S., Brangwynne C.P. Chromatin mechanics dictates subdiffusion and coarsening dynamics of embedded condensates. Nat. Phys. 2020; 17:531–538.

[10] Eeftens J.M. , Kapoor M., Michieletto D., Brangwynne C.P. Polycomb condensates can promote epigenetic marks but are not required for sustained chromatin compaction. Nat. Commun. 2021; 12:5888. 34620850 10.1038/s41467-021-26147-5 PMC8497513

[11] McSwiggen D.T. , Mir M., Darzacq X., Tjian R. Evaluating phase separation in live cells: diagnosis, caveats, and functional consequences. Genes Dev. 2019; 33:1619–1634. 31594803 10.1101/gad.331520.119 PMC6942051

[12] Shaban H.A. , Barth R., Recoules L., Bystricky K. Hi-D: nanoscale mapping of nuclear dynamics in single living cells. Genome Biol. 2020; 21:95. 32312289 10.1186/s13059-020-02002-6 PMC7168861

[13] Chen B. , Gilbert L.A., Cimini B.A., Schnitzbauer J., Zhang W., Li G.-W., Park J., Blackburn E.H., Weissman J.S., Qi L.S. et al. Dynamic imaging of genomic loci in living human cells by an optimized CRISPR/Cas system. Cell. 2013; 155:1479–1491. 24360272 10.1016/j.cell.2013.12.001 PMC3918502

[14] Chen B. , Hu J., Almeida R., Liu H., Balakrishnan S., Covill-Cooke C., Lim W.A., Huang B. Expanding the CRISPR imaging toolset with Staphylococcus aureus Cas9 for simultaneous imaging of multiple genomic loci. Nucleic Acids Res. 2016; 44:e75. 26740581 10.1093/nar/gkv1533 PMC4856973

[15] Gu B. , Swigut T., Spencley A., Bauer M.R., Chung M., Meyer T., Wysocka J. Transcription-coupled changes in nuclear mobility of mammalian cis-regulatory elements. Science. 2018; 359:1050–1055. 29371426 10.1126/science.aao3136 PMC6590518

[16] Shao S. , Chang L., Sun Y., Hou Y., Fan X., Sun Y. Multiplexed sgRNA expression allows versatile single nonrepetitive DNA labeling and endogenous gene regulation. ACS Synth. Biol. 2018; 7:176–186. 28849913 10.1021/acssynbio.7b00268

[17] Normanno D. , Boudarène L., Dugast-Darzacq C., Chen J., Richter C., Proux F., Bénichou O., Voituriez R., Darzacq X., Dahan M. Probing the target search of DNA-binding proteins in mammalian cells using TetR as model searcher. Nat. Commun. 2015; 6:7357. 26151127 10.1038/ncomms8357 PMC4507003

[18] Du M. , Kodner S., Bai L. Enhancement of LacI binding in vivo. Nucleic Acids Res. 2019; 47:9609–9618. 31396617 10.1093/nar/gkz698 PMC6765135

[19] Ma H. , Tu L.-C., Naseri A., Huisman M., Zhang S., Grunwald D., Pederson T. Multiplexed labeling of genomic loci with dCas9 and engineered sgRNAs using CRISPRainbow. Nat. Biotechnol. 2016; 34:528–530. 27088723 10.1038/nbt.3526 PMC4864854

[20] Ma H. , Naseri A., Reyes-Gutierrez P., Wolfe S.A., Zhang S., Pederson T. Multicolor CRISPR labeling of chromosomal loci in human cells. Proc. Natl. Acad. Sci. 2015; 112:3002–3007. 25713381 10.1073/pnas.1420024112 PMC4364232

[21] Ma H. , Tu L.-C., Naseri A., Chung Y.-C., Grunwald D., Zhang S., Pederson T. CRISPR-Sirius: RNA scaffolds for signal amplification in genome imaging. Nat. Methods. 2018; 15:928–931. 30377374 10.1038/s41592-018-0174-0 PMC6252086

[22] Qin P. , Parlak M., Kuscu C., Bandaria J., Mir M., Szlachta K., Singh R., Darzacq X., Yildiz A., Adli M. Live cell imaging of low- and non-repetitive chromosome loci using CRISPR-Cas9. Nat. Commun. 2017; 8:14725. 28290446 10.1038/ncomms14725 PMC5424063

[23] Manley S. , Gillette J.M., Patterson G.H., Shroff H., Hess H.F., Betzig E., Lippincott-Schwartz J. High-density mapping of single-molecule trajectories with photoactivated localization microscopy. Nat. Methods. 2008; 5:155–157. 18193054 10.1038/nmeth.1176

[24] Iino R. , Koyama I., Kusumi A. Single molecule imaging of green fluorescent proteins in living cells: E-cadherin forms oligomers on the free cell surface. Biophys. J. 2001; 80:2667–2677. 11371443 10.1016/S0006-3495(01)76236-4 PMC1301454

[25] Rust M.J. , Bates M., Zhuang X. Stochastic optical reconstruction microscopy (STORM) provides sub-diffraction-limit image resolution. Nat. Methods. 2006; 3:793–795. 16896339 10.1038/nmeth929 PMC2700296

[26] Betzig E. , Patterson G.H., Sougrat R., Lindwasser O.W., Olenych S., Bonifacino J.S., Davidson M.W., Lippincott-Schwartz J., Hess H.F. Imaging intracellular fluorescent proteins at nanometer resolution. Science. 2006; 313:1642–1645. 16902090 10.1126/science.1127344

[27] Knight S.C. , Xie L., Deng W., Guglielmi B., Witkowsky L.B., Bosanac L., Zhang E.T., El Beheiry M., Masson J.-B., Dahan M. et al. Dynamics of CRISPR-Cas9 genome interrogation in living cells. Science. 2015; 350:823–826. 26564855 10.1126/science.aac6572

[28] Martens K.J.A. , van Beljouw S.P.B., van der Els S., Vink J.N.A., Baas S., Vogelaar G.A., Brouns S.J.J., van Baarlen P., Kleerebezem M., Hohlbein J. Visualisation of dCas9 target search in vivo using an open-microscopy framework. Nat. Commun. 2019; 10:3552. 31391532 10.1038/s41467-019-11514-0 PMC6685946

[29] Zhu Y. , Li P., Beuzer P., Tong Z., Watters R., Lv D., Murre C., Cang H. CRISPR/Cas9 for photoactivated localization microscopy (PALM). 2014; arXiv doi:26 March 2014, preprint: not peer reviewed https://arxiv.org/abs/1403.6738.

[30] Jones D.L. , Leroy P., Unoson C., Fange D., Ćurić V., Lawson M.J., Elf J. Kinetics of dCas9 target search in Escherichia coli. Science. 2017; 357:1420–1424. 28963258 10.1126/science.aah7084 PMC6150439

[31] Neguembor M.V. , Sebastian-Perez R., Aulicino F., Gomez-Garcia P.A., Cosma M.P., Lakadamyali M. Po)STAC (Polycistronic Suntag modified CRISPR) enables live-cell and fixed-cell super-resolution imaging of multiple genes. Nucleic Acids Res. 2018; 46:e30. 29294098 10.1093/nar/gkx1271 PMC5861460

[32] Jain S. , Shukla S., Yang C., Zhang M., Fatma Z., Lingamaneni M., Abesteh S., Lane S.T., Xiong X., Wang Y. et al. TALEN outperforms Cas9 in editing heterochromatin target sites. Nat. Commun. 2021; 12:606. 33504770 10.1038/s41467-020-20672-5 PMC7840734

[33] Lerner J. , Gomez-Garcia P.A., McCarthy R.L., Liu Z., Lakadamyali M., Zaret K.S. Two-Parameter mobility assessments discriminate diverse regulatory factor behaviors in chromatin. Mol. Cell. 2020; 79:677–688. 32574554 10.1016/j.molcel.2020.05.036 PMC7483934

[34] Inavalli V.V.G.K. , Lenz M.O., Butler C., Angibaud J., Compans B., Levet F., Tønnesen J., Rossier O., Giannone G., Thoumine O. et al. A super-resolution platform for correlative live single-molecule imaging and STED microscopy. Nat. Methods. 2019; 16:1263–1268. 31636458 10.1038/s41592-019-0611-8

[35] Weiss L.E. , Milenkovic L., Yoon J., Stearns T., Moerner W.E. Motional dynamics of single Patched1 molecules in cilia are controlled by Hedgehog and cholesterol. Proc. Natl. Acad. Sci. USA. 2019; 116:5550–5557. 30819883 10.1073/pnas.1816747116 PMC6431229

[36] Milenkovic L. , Weiss L.E., Yoon J., Roth T.L., Su Y.S., Sahl S.J., Scott M.P., Moerner W.E. Single-molecule imaging of Hedgehog pathway protein Smoothened in primary cilia reveals binding events regulated by Patched1. Proc. Natl. Acad. Sci. USA. 2015; 112:8320–8325. 26100903 10.1073/pnas.1510094112 PMC4500289

[37] Basu S. , Shukron O., Hall D., Parruto P., Ponjavic A., Shah D., Boucher W., Lando D., Zhang W., Reynolds N., Sober L.H. et al. Live-cell 3D single-molecule tracking reveals how NuRD modulates enhancer dynamics. 2021; bioRxiv doi:19 July 2021, preprint: not peer reviewed 10.1101/2020.04.03.003178.

[38] Donovan B.T. , Huynh A., Ball D.A., Patel H.P., Poirier M.G., Larson D.R., Ferguson M.L., Lenstra T.L. Live-cell imaging reveals the interplay between transcription factors, nucleosomes, and bursting. EMBO J. 2019; 38:e100809. 31101674 10.15252/embj.2018100809 PMC6576174

[39] Wang S. , Hao Y., Zhang L., Wang F., Li J., Wang L., Fan C. Multiplexed superresolution CRISPR imaging of chromatin in living cells. CCS Chem. 2019; 1:278–285.

[40] McSwiggen D.T. , Hansen A.S., Teves S.S., Marie-Nelly H., Hao Y., Heckert A.B., Umemoto K.K., Dugast-Darzacq C., Tjian R., Darzacq X. Evidence for DNA-mediated nuclear compartmentalization distinct from phase separation. Elife. 2019; 8:e47098. 31038454 10.7554/eLife.47098 PMC6522219

[41] Li J. , Hsu A., Hua Y., Wang G., Cheng L., Ochiai H., Yamamoto T., Pertsinidis A. Single-gene imaging links genome topology, promoter-enhancer communication and transcription control. Nat. Struct. Mol. Biol. 2020; 27:1032–1040. 32958948 10.1038/s41594-020-0493-6 PMC7644657

[42] Schmidt J.C. , Zaug A.J., Cech T.R. Live cell imaging reveals the dynamics of telomerase recruitment to telomeres. Cell. 2016; 166:1188–1197. 27523609 10.1016/j.cell.2016.07.033 PMC5743434

[43] Hell S.W. , Wichmann J. Breaking the diffraction resolution limit by stimulated emission: stimulated-emission-depletion fluorescence microscopy. Opt. Lett. 1994; 19:780–782. 19844443 10.1364/ol.19.000780

[44] Westphal V. , Lauterbach M.A., Nicola A.D., Hell S.W. Dynamic far-field fluorescence nanoscopy. New J. Phys. 2007; 9:435–435.

[45] Westphal V. , Rizzoli S.O., Lauterbach M.A., Kamin D., Jahn R., Hell S.W. Video-Rate far-field optical nanoscopy dissects synaptic vesicle movement. Science. 2008; 320:246–249. 18292304 10.1126/science.1154228

[46] Gustafsson M.G.L. Surpassing the lateral resolution limit by a factor of two using structured illumination microscopy. J. Microsc. 2000; 198:82–87. 10810003 10.1046/j.1365-2818.2000.00710.x

[47] Gustafsson M.G.L. , Shao L., Carlton P.M., Wang C.J.R., Golubovskaya I.N., Cande W.Z., Agard D.A., Sedat J.W. Three-Dimensional resolution doubling in wide-field fluorescence microscopy by structured illumination. Biophys. J. 2008; 94:4957–4970. 18326650 10.1529/biophysj.107.120345 PMC2397368

[48] Adhikari S. , Moscatelli J., Smith E.M., Banerjee C., Puchner E.M. Single-molecule localization microscopy and tracking with red-shifted states of conventional BODIPY conjugates in living cells. Nat. Commun. 2019; 10:3400. 31363088 10.1038/s41467-019-11384-6 PMC6667493

[49] Smith E.M. , Gautier A., Puchner E.M. Single-Molecule localization microscopy with the fluorescence-activating and absorption-shifting tag (FAST) system. ACS Chem. Biol. 2019; 14:1115–1120. 31083964 10.1021/acschembio.9b00149 PMC8608280

[50] Banerjee C. , Mehra D., Song D., Mancebo A., Kim D.-H., Puchner E.M. ULK1 forms distinct oligomeric states and nanoscopic morphologies during autophagy initiation. 2020; bioRxiv doi:07 December 2020, preprint: not peer reviewed 10.1101/2020.07.03.187336. PMC10541014 37774021

[51] Mancebo A. , DeMars L., Ertsgaard C.T., Puchner E.M. Precisely calibrated and spatially informed illumination for conventional fluorescence and improved PALM imaging applications. Methods Appl. Fluoresc. 2020; 8:025004. 31995796 10.1088/2050-6120/ab716a PMC8609923

[52] Gómez-García P.A. , Portillo-Ledesma S., Neguembor M.V., Pesaresi M., Oweis W., Rohrlich T., Wieser S., Meshorer E., Schlick T., Cosma M.P. et al. Mesoscale modeling and single-nucleosome tracking reveal remodeling of clutch folding and dynamics in stem cell differentiation. Cell Rep. 2021; 34:108614. 33440158 10.1016/j.celrep.2020.108614 PMC7842188

[53] Thompson R.E. , Larson D.R., Webb W.W. Precise nanometer localization analysis for individual fluorescent probes. Biophys. J. 2002; 82:2775–2783. 11964263 10.1016/S0006-3495(02)75618-X PMC1302065

[54] Adhikari S. , Moscatelli J., Puchner E.M. Quantitative live-cell PALM reveals nanoscopic Faa4 redistributions and dynamics on lipid droplets during metabolic transitions of yeast. Mol. Biol. Cell. 2021; 32:1565–1578. 34161133 10.1091/mbc.E20-11-0695 PMC8351750

[55] Karslake J.D. , Donarski E.D., Shelby S.A., Demey L.M., DiRita V.J., Veatch S.L., Biteen J.S. SMAUG: Analyzing single-molecule tracks with nonparametric Bayesian statistics. Methods. 2021; 193:16–26. 32247784 10.1016/j.ymeth.2020.03.008 PMC7529709

[56] Metzler R. , Jeon J.-H., Cherstvy A.G., Barkai E. Anomalous diffusion models and their properties: non-stationarity, non-ergodicity, and ageing at the centenary of single particle tracking. Phys. Chem. Chem. Phys. 2014; 16:24128–24164. 25297814 10.1039/c4cp03465a

[57] Amitai A. , Holcman D. Polymer physics of nuclear organization and function. Physics Reports. 2017; 678:1–83.

[58] Kepten E. , Weron A., Sikora G., Burnecki K., Garini Y. Guidelines for the fitting of anomalous diffusion mean square displacement graphs from single particle tracking experiments. PLOS ONE. 2015; 10:e0117722. 25680069 10.1371/journal.pone.0117722 PMC4334513

[59] von Diezmann L. , Rog O. Single-Molecule tracking of chromatin-associated proteins in the C. elegans Gonad. J. Phys. Chem. B. 2021; 125:6162–6170. 34097417 10.1021/acs.jpcb.1c03040 PMC9045689

[60] Backlund M.P. , Joyner R., Weis K., Moerner W.E. Correlations of three-dimensional motion of chromosomal loci in yeast revealed by the double-helix point spread function microscope. Mol. Biol. Cell. 2014; 25:3619–3629. 25318676 10.1091/mbc.E14-06-1127 PMC4230621

[61] Backlund M.P. , Joyner R., Moerner W.E. Chromosomal locus tracking with proper accounting of static and dynamic errors. Phys. Rev. E Stat. Nonlin. Soft Matter Phys. 2015; 91:062716. 26172745 10.1103/PhysRevE.91.062716 PMC4533921

[62] Hansen A.S. , Woringer M., Grimm J.B., Lavis L.D., Tjian R., Darzacq X. Robust model-based analysis of single-particle tracking experiments with Spot-On. Elife. 2018; 7:e33125. 29300163 10.7554/eLife.33125 PMC5809147

[63] Zhen C.Y. , Tatavosian R., Huynh T.N., Duc H.N., Das R., Kokotovic M., Grimm J.B., Lavis L.D., Lee J., Mejia F.J. et al. Live-cell single-molecule tracking reveals co-recognition of H3K27me3 and DNA targets polycomb Cbx7-PRC1 to chromatin. Elife. 2016; 5:e17667. 27723458 10.7554/eLife.17667 PMC5056789

[64] Cho W.-K. , Jayanth N., English B.P., Inoue T., Andrews J.O., Conway W., Grimm J.B., Spille J.-H., Lavis L.D., Lionnet T. et al. RNA polymerase II cluster dynamics predict mRNA output in living cells. Elife. 2016; 5:e13617. 27138339 10.7554/eLife.13617 PMC4929003

[65] Wang A.S. , Chen L.C., Wu R.A., Hao Y., McSwiggen D.T., Heckert A.B., Richardson C.D., Gowen B.G., Kazane K.R., Vu J.T. et al. The histone chaperone FACT induces Cas9 Multi-turnover behavior and modifies genome manipulation in human cells. Mol. Cell. 2020; 79:221–233. 32603710 10.1016/j.molcel.2020.06.014 PMC7398558

[66] Ma H. , Tu L.-C., Naseri A., Huisman M., Zhang S., Grunwald D., Pederson T. CRISPR-Cas9 nuclear dynamics and target recognition in living cells. J. Cell Biol. 2016; 214:529–537. 27551060 10.1083/jcb.201604115 PMC5004447

[67] Vancevska A. , Douglass K.M., Pfeiffer V., Manley S., Lingner J. The telomeric DNA damage response occurs in the absence of chromatin decompaction. Genes Dev. 2017; 31:567–577. 28381410 10.1101/gad.294082.116 PMC5393052

[68] Doksani Y. , Wu J.Y., de Lange T., Zhuang X. Super-resolution fluorescence imaging of telomeres reveals TRF2-dependent T-loop formation. Cell. 2013; 155:345–356. 24120135 10.1016/j.cell.2013.09.048 PMC4062873

[69] Timashev L.A. , Babcock H., Zhuang X., de Lange T. The DDR at telomeres lacking intact shelterin does not require substantial chromatin decompaction. Genes Dev. 2017; 31:578–589. 28381412 10.1101/gad.294108.116 PMC5393053

[70] Eaton J.A. , Zidovska A. Structural and dynamical signatures of local DNA damage in live cells. Biophys. J. 2020; 118:2168–2180. 31818467 10.1016/j.bpj.2019.10.042 PMC7202935

[71] Nozaki T. , Imai R., Tanbo M., Nagashima R., Tamura S., Tani T., Joti Y., Tomita M., Hibino K., Kanemaki M.T. et al. Dynamic organization of chromatin domains revealed by super-resolution live-cell imaging. Mol. Cell. 2017; 67:282–293. 28712725 10.1016/j.molcel.2017.06.018

[72] Nagashima R. , Hibino K., Ashwin S.S., Babokhov M., Fujishiro S., Imai R., Nozaki T., Tamura S., Tani T., Kimura H. et al. Single nucleosome imaging reveals loose genome chromatin networks via active RNA polymerase II. J. Cell Biol. 2019; 218:1511–1530. 30824489 10.1083/jcb.201811090 PMC6504897

[73] Ashwin S.S. , Nozaki T., Maeshima K., Sasai M. Organization of fast and slow chromatin revealed by single-nucleosome dynamics. Proc. Natl. Acad. Sci. 2019; 116:19939–19944. 31527274 10.1073/pnas.1907342116 PMC6778247

[74] Berglund A.J. Statistics of camera-based single-particle tracking. Phys. Rev. E. 2010; 82:011917. 10.1103/PhysRevE.82.011917 20866658

[75] Michalet X. , Berglund A.J. Optimal diffusion coefficient estimation in single-particle tracking. Phys. Rev. E. 2012; 85:061916. 10.1103/PhysRevE.85.061916 PMC4917385 23005136

[76] Shechtman Y. , Gustavsson A.-K., Petrov P.N., Dultz E., Lee M.Y., Weis K., Moerner W.E. Observation of live chromatin dynamics in cells via 3D localization microscopy using Tetrapod point spread functions. Biomed. Opt. Express. 2017; 8:5735–5748. 29296501 10.1364/BOE.8.005735 PMC5745116

[77] Nehme E. , Freedman D., Gordon R., Ferdman B., Weiss L.E., Alalouf O., Naor T., Orange R., Michaeli T., Shechtman Y. DeepSTORM3D: dense 3D localization microscopy and PSF design by deep learning. Nat. Methods. 2020; 17:734–740. 32541853 10.1038/s41592-020-0853-5 PMC7610486

[78] Gustavsson A.-K. , Ghosh R.P., Petrov P.N., Liphardt J., Moerner W.E. Fast and parallel nanoscale 3D tracking of heterogeneous mammalian chromatin dynamics. MBoC. 2022; 10.1091/mbc.E21-10-0514. PMC9265149 35352962

[79] Isaac R.S. , Jiang F., Doudna J.A., Lim W.A., Narlikar G.J., Almeida R. Nucleosome breathing and remodeling constrain CRISPR-Cas9 function. Elife. 2016; 5:e13450. 27130520 10.7554/eLife.13450 PMC4880442

[80] Horlbeck M.A. , Witkowsky L.B., Guglielmi B., Replogle J.M., Gilbert L.A., Villalta J.E., Torigoe S.E., Tjian R., Weissman J.S. Nucleosomes impede Cas9 access to DNA in vivo and in vitro. Elife. 2016; 5:e12677. 26987018 10.7554/eLife.12677 PMC4861601

