# Supplementary material for: Characterizing locus specific chromatin structure and dynamics with correlative conventional and super-resolution imaging in living cells
Source: Nucleic Acids Res. 2022 May 7;50(13):e78. doi: 10.1093/nar/gkac314 (PMC9303368; doi:10.1093/nar/gkac314)
Supplement: gkac314_Supplemental_Files [file gkac314_supplemental_files.zip › Supplemental Figures_R3.pdf]

Supplementary Information:

Characterizing Locus Specific Chromatin Structure and Dynamics with Correlative Conventional and Super Resolution imaging in living cells

Dushyant Mehra[1,2], Santosh Adhikari[1], Chiranjib Banerjee[1], Elias M. Puchner[1*]

[1] School of Physics and Astronomy, University of Minnesota, Minneapolis MN

[2] Department of Physiology and Biomedical Engineering, Mayo Clinic, Rochester MN

* Corresponding Author: epuchner@umn.edu

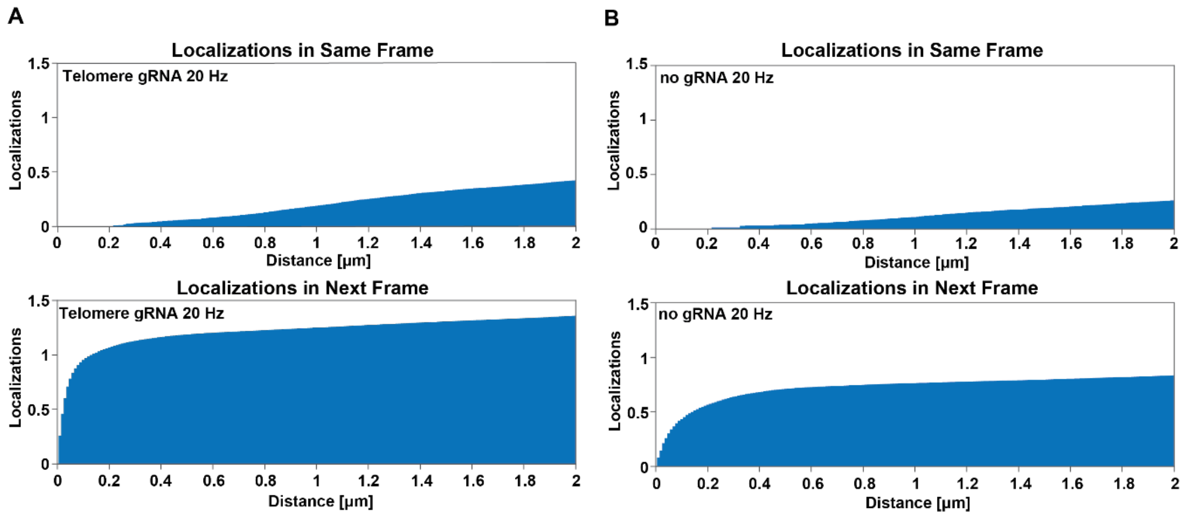

Supplementary Figure 1: Likelihood of False Linking in Single Molecule Tracking.

The pair correlation function quantifies how many localizations are found around each localization within a given distance in the same frame (top) or next frame (bottom) **A)** For MCP-HaloTag tracking in the presence of telomere gRNA, on average two localizations are within a distance of 5.05 μm in the same frame (indicating the distance threshold for false linking) and 120 nm in the subsequent frame (indicating the distance of the same molecule from its previous location). Only 0.034 localizations are within the 480 nm linking threshold in the same frame. This indicates a 3.4% chance of false linking using the 480 nm linking threshold. **B)** For MCP-HaloTag tracking with no gRNA, on average two localizations are within a distance of 11.32 μm in the same frame and 3.97 μm in the subsequent frame. 0.022 localizations are found within the 480 nm linking threshold in the same frame. This indicates a 2.2% chance of false linking using the 480 nm linking threshold. Data was collected from N = 5 telomere gRNA at 20 Hz and N = 5 no gRNA cells at 20 Hz.

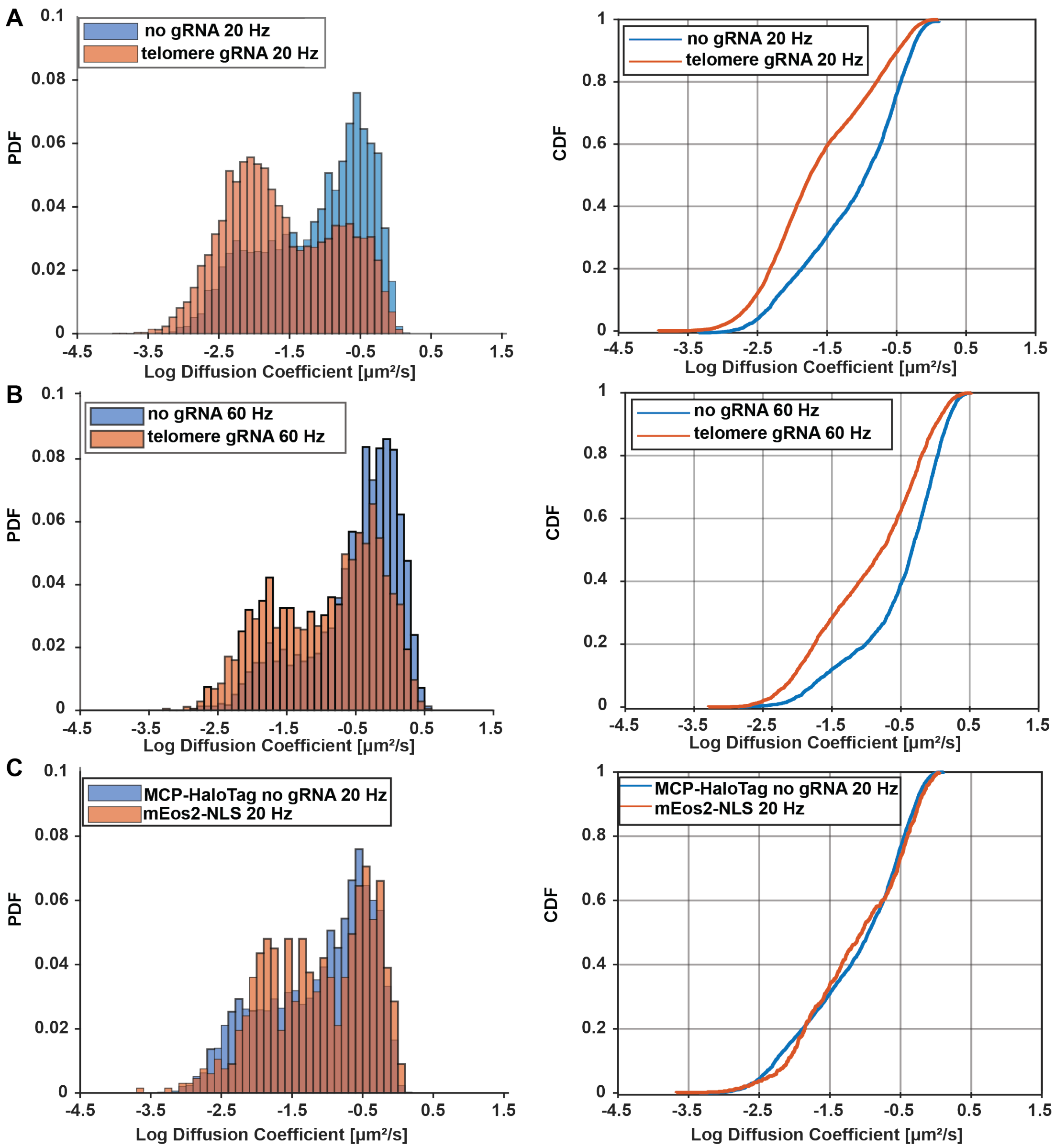

Supplementary Figure 2: Comparison of diffusion coefficient distributions of data recorded at 20 Hz and 60 Hz frame rate. A) Probability density function (left) and cumulative probability density function (right) of the diffusion coefficient distribution of MCP-HaloTag with and without telomere gRNA recorded at 20 Hz frame rate (same data as presented in Figure 1C). **B)** Probability density function (left) and cumulative probability density function (right) of the diffusion coefficient distribution of MCP-HaloTag with and without telomere gRNA recorded at 60 Hz frame rate (n = 4875 traces with telomere gRNA and N = 3518 traces without gRNA from N = 3 cells for each case). There was no statistically significant difference between the distributions recorded at 20 Hz and 60 Hz (Kologmorov-Smirnov Test: P = 0.65 and P = 0.72). **C)** Probability density function (left) and cumulative probability density function (right) of mEos2-NLS (N = 2936 traces from N = 3 cells) and MCP-HaloTag recorded at 20 Hz (same as MCP-HaloTag data presented in Figure 1C and Supplementary Figure 2A). There was no statistically significant difference between the distributions of MCP-HaloTag and mEos2-NLS (Kologmorov-Smirnov Test P = 0.62).

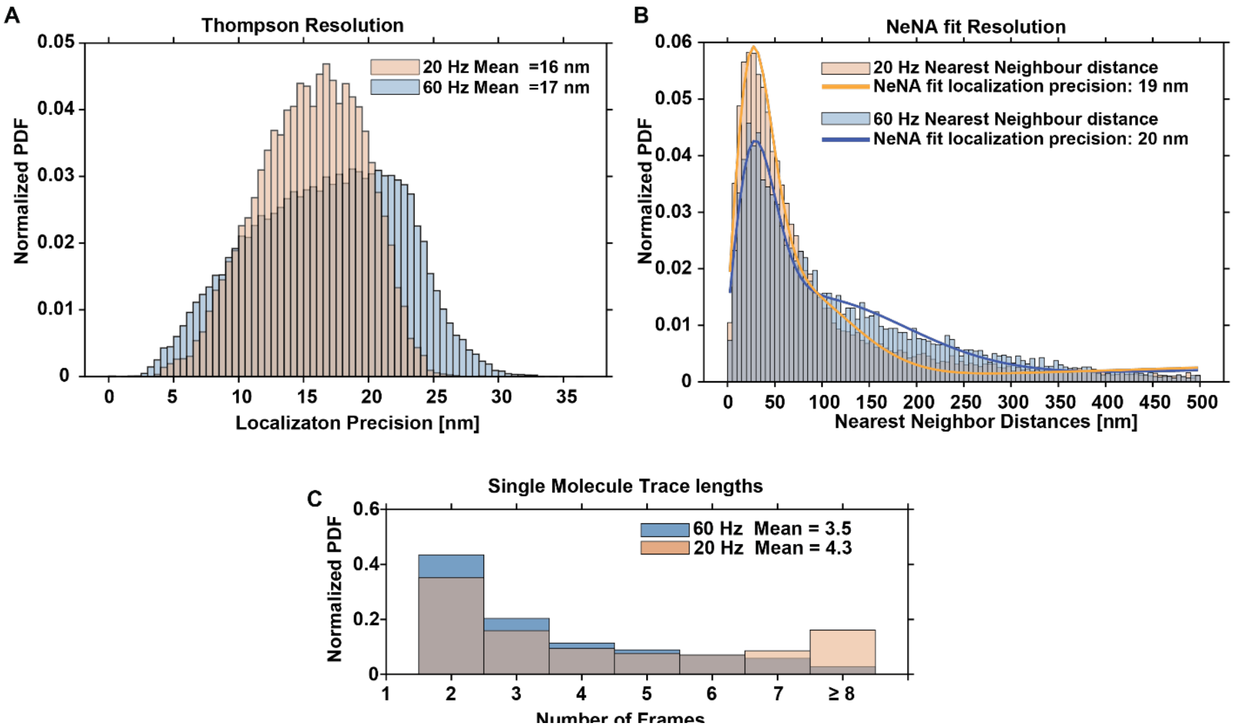

Supplementary Figure 3: Single molecule localization error and trace length comparison at 20Hz and 60Hz frame rate

A) Probability density function of the PALM localization precision at 20Hz and 60Hz frame rates. The localization precision was calculated using the Thompson resolution formula (1). Though this formula underestimates the true localization precision, it is useful for a relative localization precision comparison between the 20Hz and 60Hz PALM movies (2). Both the mean and median localization precision was lower during 20Hz imaging than 60Hz imaging. **B)** Probability density function of the PALM localization precision at 20Hz and 60Hz frame rates estimated with the nearest neighbor method from reference (3) (**C)** Probability density function of the single molecule trajectory lengths obtained from 20Hz and 60Hz PALM data. The mean trace length is higher for PALM data taken at 20Hz than at 60Hz. This indicates that taking PALM data at 20Hz is more advantageous than at 60Hz because of the higher localization precision and the longer single molecule trajectories.

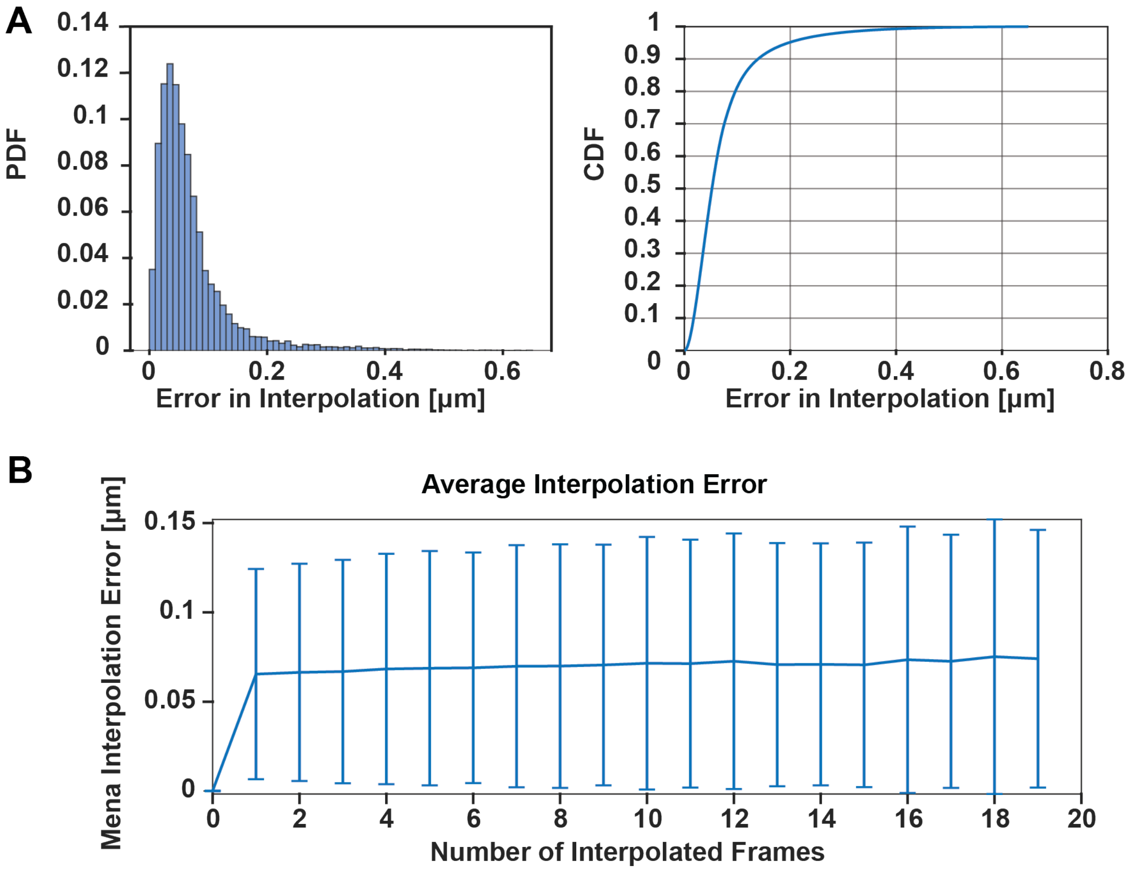

Supplementary Figure 4: Interpolation Error of GFP Cluster Tracking

A) Probability density function (left) and cumulative density function (right) of the interpolation error of telomere clusters. Telomeres were continuously imaged and tracked with the conventional fluorescence signal of GFP at 20 Hz for 200 seconds. To mimic the interpolation of the correlative conventional and PALM imaging data in which the telomere GFP signal is imaged and localized every 10th frame, the localizations of telomeres were linearly interpolated between every 10th frame and compared to the actual localizations in the remaining frames. The distances between the centers of the interpolated positions and the actual positions were calculated to estimate the interpolation error. **B)** Average error of the interpolation across a varying number of interpolated frames. The small increase in the error if localizations are interpolated across a larger number of frames indicates that the error is not dominated by the interpolation but rather by the localization uncertainty itself. The error bars represent the standard deviation of the mean interpolation error distribution. Data represents 181 telomeres from N=5 different cells.

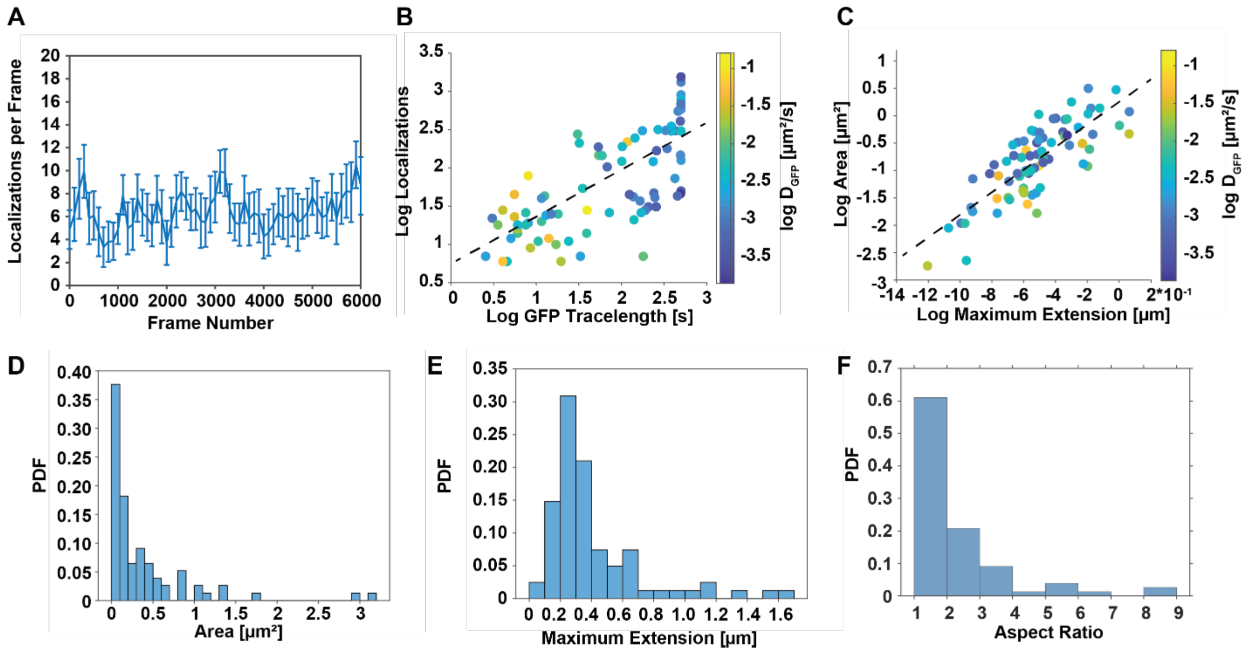

Supplementary Figure 5: Normalization of the number of localizations by the observation time of telomeres and telomere size quantification.

A) The detected number of PALM localizations of MCP-HaloTag per frame is constant for the duration of the movie. **B)** The number of localizations detected for each telomere must be normalized by the time a telomere stayed in focus in order to correct for differences in the number of detected localizations. **C)** Comparison between the area of telomeres determined from motion corrected single molecule localizations and their maximum distance. While there is a correlation between the telomere area and their maximum extension (correlation coefficient = 0.82), deviations indicate irregular shapes. **D)** Histogram of telomere areas **E)** maximum extension and **F**) aspect ratio from elliptical fitting. All data displayed in this figure is from N = 5 cells and N = 81 telomere clusters.

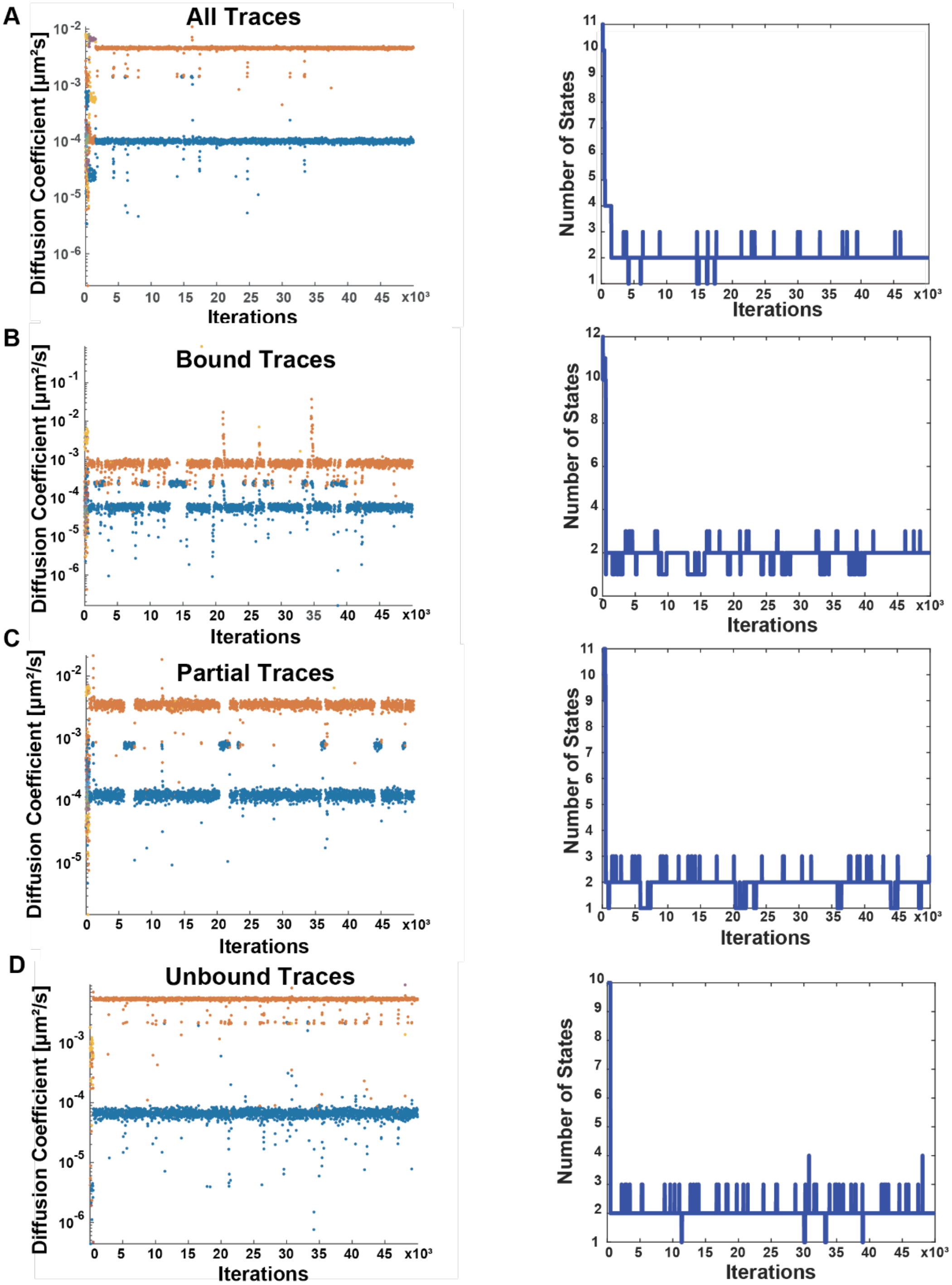

Supplementary Figure 6: SMAUG algorithm convergence analysis

A)-D) Left: Diffusion coefficients of mobility states per iteration for all traces, bound traces, unbound traces, and partial traces identified by motion correction. **Right**: Number of the mobility states of the SMAUG algorithm identified for each of the 50000 iterations. These plots highlight the quick convergence of the SMAUG algorithm to two mobility states and to the associated diffusion coefficients. The occasional jumps to one, three and four mobility states show parameter space sampling of the algorithm.

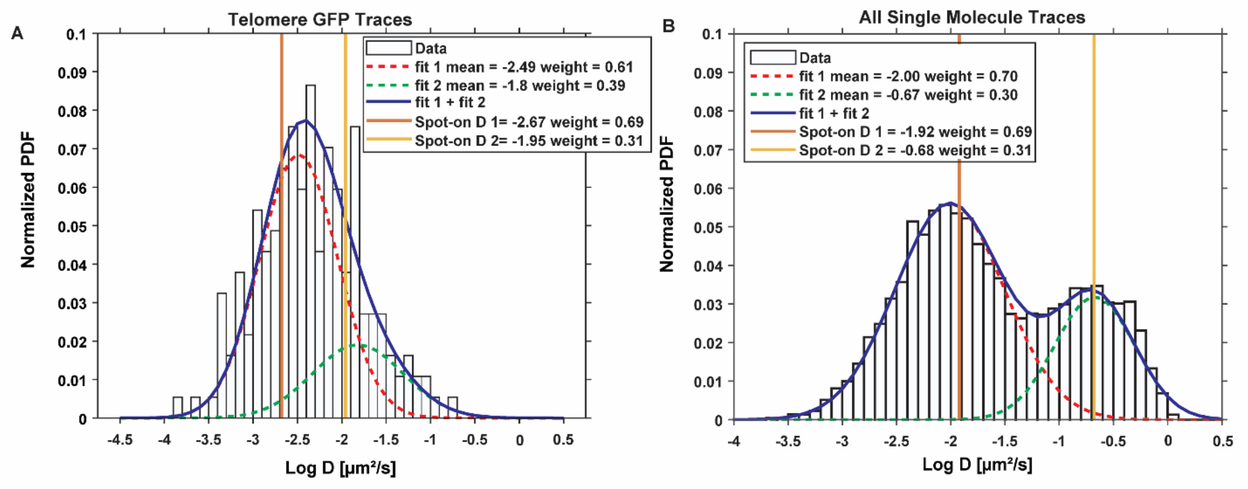

Supplementary Figure 7: Spot-on analysis and Gaussian mixture model comparison of telomere and single molecule traces

A) The normalized probability distribution of telomere diffusion coefficients from linear MSD fitting was fitted to a two state Gaussian mixture model and analyzed by spot-on analysis using a 2 state model. The red and green Gaussian distribution represent the sub-populations obtained from Gaussian fitting while the blue line represents the sum of both Gaussian fits. Orange and yellow lines represent the diffusion coefficient values of the bound and unbound sub populations identified by spot-on analysis. Diffusion coefficient values identified by Gaussian mixture model fitting and spot-on analysis are reported in the figure legend. **B)** The normalized probability distribution of all single molecule diffusion coefficients was fitted to a two state Gaussian mixture model and analyzed by spot-on analysis using a 2 state model. The green and red Gaussian distribution represent the respective sub-populations obtained from Gaussian fitting while the blue line represents the sum of both Gaussian fits. Orange and yellow lines represent the diffusion coefficients of the respective bound and unbound populations identified by the spot-on analysis. Diffusion coefficient values and weight fractions of sub populations identified by Gaussian mixture model fitting and spot-on analysis are reported in the figure legend.

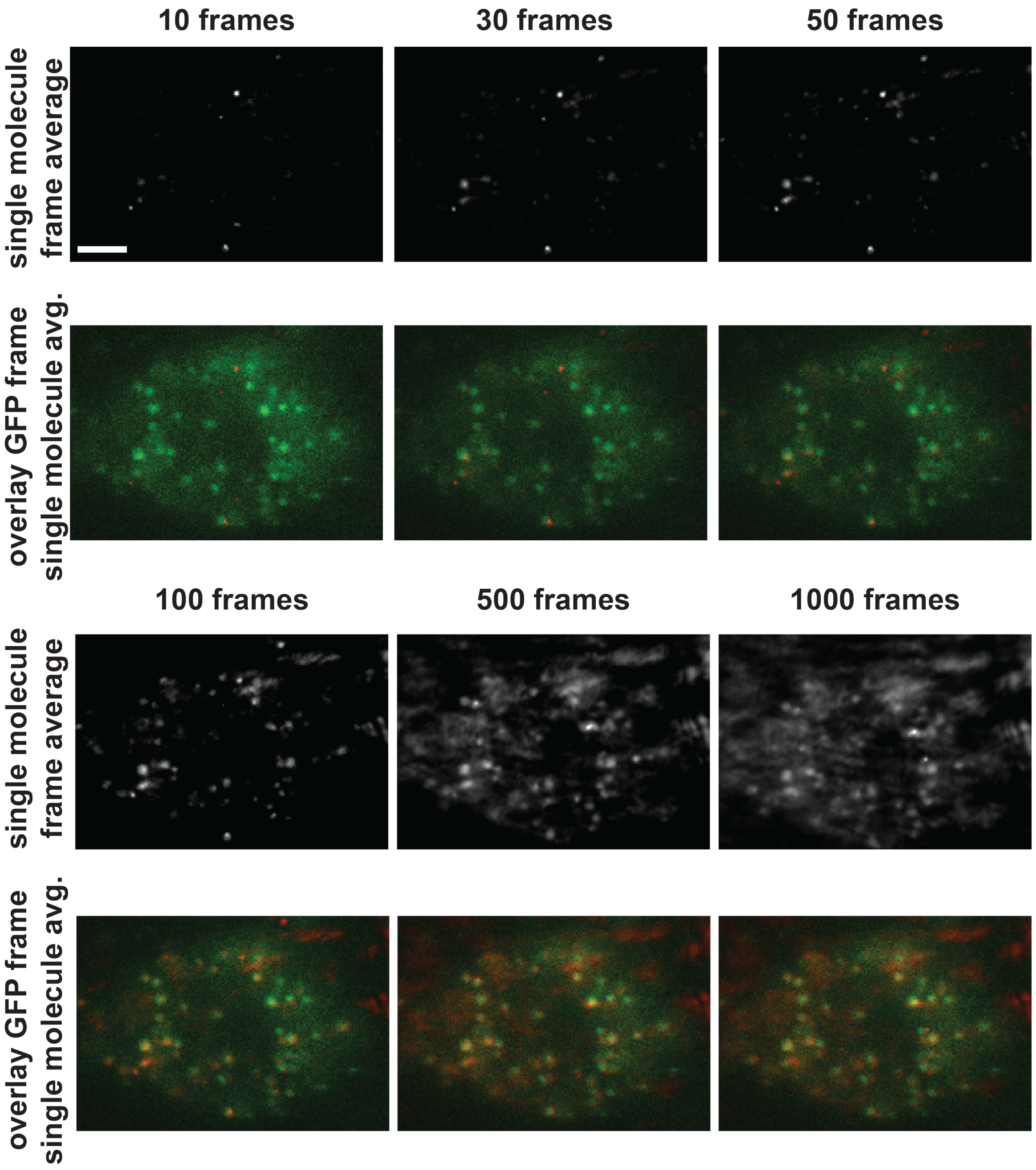

Supplementary Figure 8: Comparison of a conventional fluorescence GFP image to temporal averages of single molecule frames.

A varied number of single molecule frames were averaged (top) and superimposed to a single conventional fluorescence GFP frame taken at the same timepoint (bottom). Due to the required low photoactivation rates, averaging even up to 100 frames is not sufficient to obtain enough single molecule signals from each telomere to sample its center of mass. At even higher frame averages, smearing results from movement of telomere clusters and the presence of unbound single molecules. This indicates that the conventional fluorescence signal is needed to accurately identify the center of mass of telomere clusters.

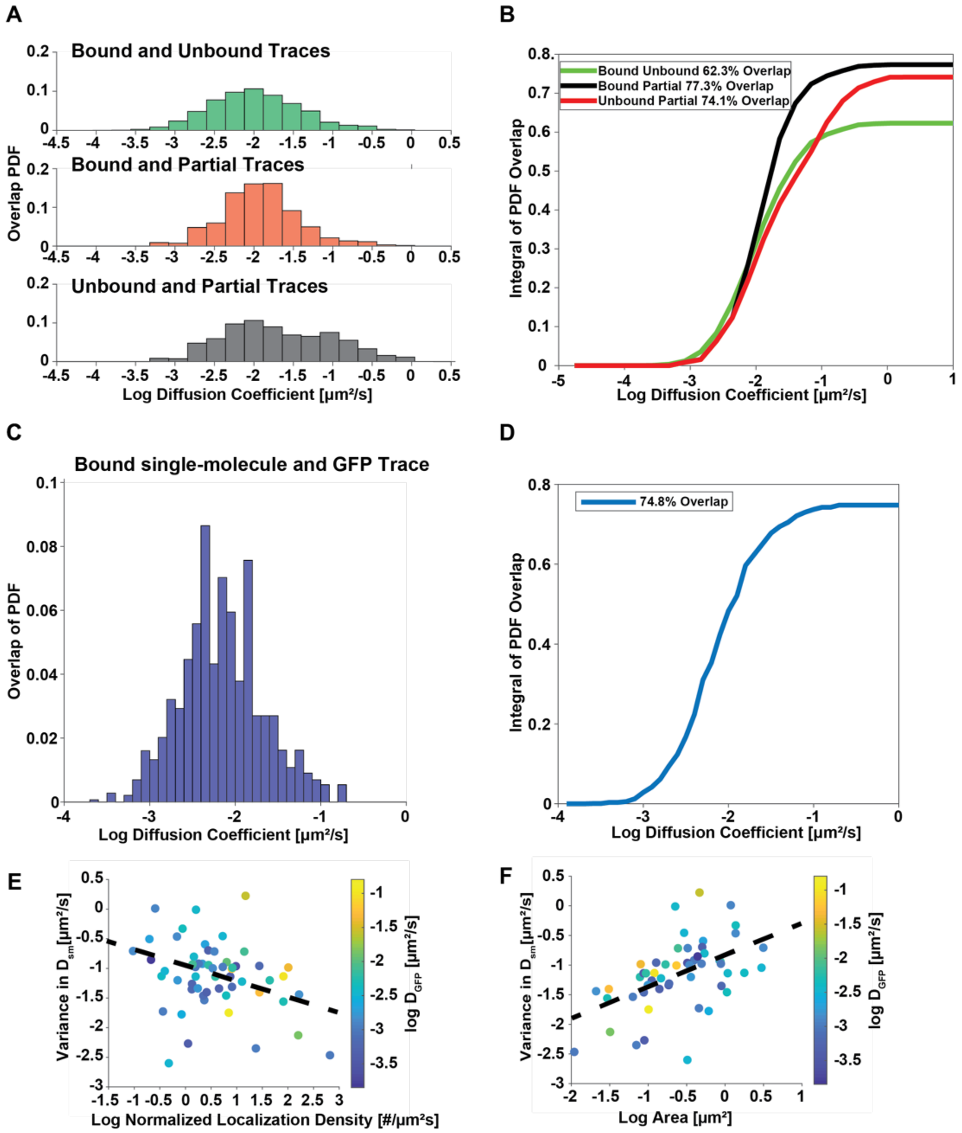

Supplemental Figure 9: Overlap of Diffusion Coefficient Distributions Calculation and Variance in the diffusion coefficients of bound MCP-HaloTag proteins.

A) Overlap integral of in the PDF of diffusion coefficient distributions shown in Figure 3A. **B)** The integral of the overlap shows the total overlap fraction as a function of the diffusion coefficient and assesses the misclassification error in when assigning a trace to a mobility state using a fixed mobility threshold. **C)** and **D)** compare the overlap in the diffusion coefficient distributions of motion corrected MCP-HaloTag traces bound to telomeres and GFP telomere traces. This plot shows that there is a high percent overlap between the two distributions but differences in both distributions remain. **E)** The variance of the diffusion coefficients of MCP-HaloTag proteins bound to individual telomeres shows a slight negative correlation (correlation coefficient = - 0.34) with the number of detected localizations. This indicates that dense telomeres exhibit less relative motion of bound MCP-HaloTag proteins. **F)** The variance of the diffusion coefficients of MCP-HaloTag proteins bound to individual telomeres shows a positive correlation with the area of telomeres (correlation coefficient = 0.47). This indicates that larger telomeres exhibit more relative motion of bound MCP-HaloTag proteins. There was no significance difference in single molecule variance across N = 5 cells as assessed by multi-way ANOVA (P = 0.25).

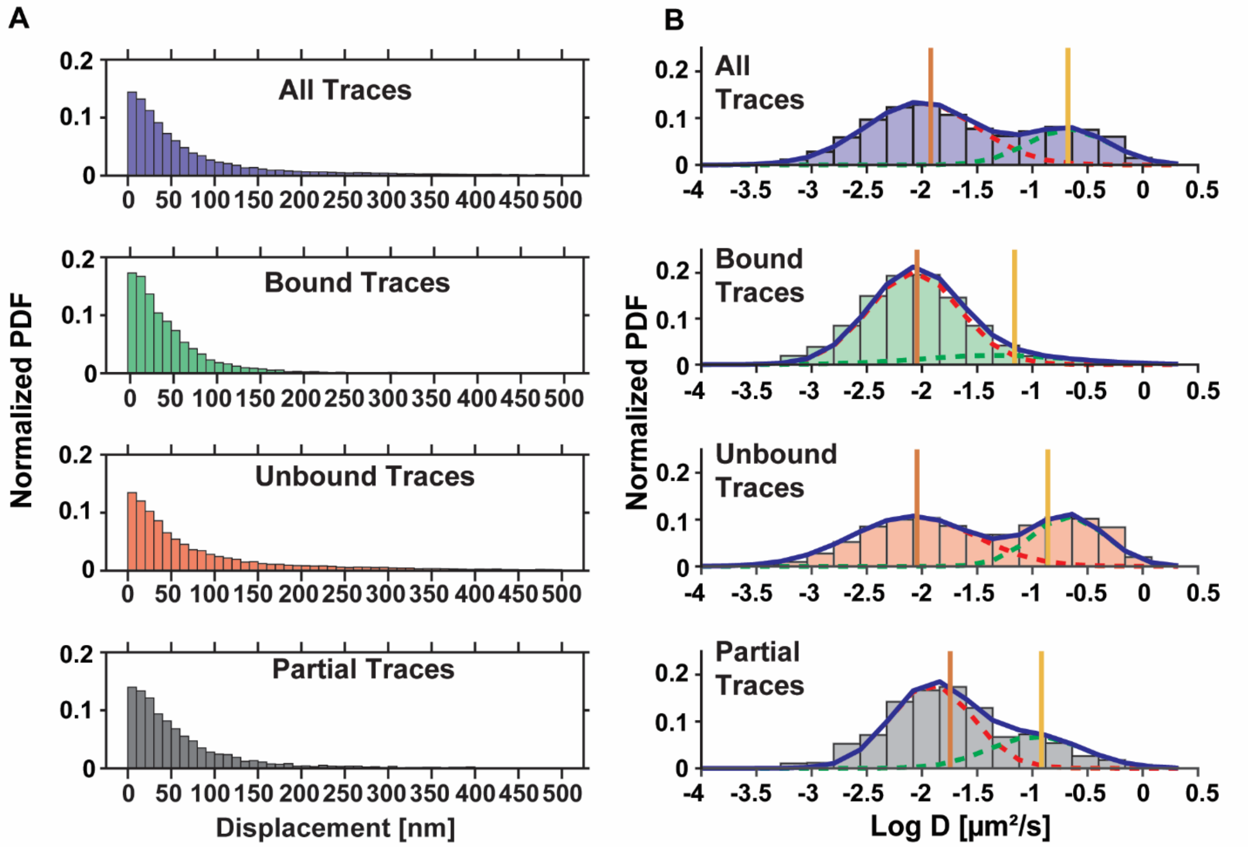

C

	Gaussian fitting				Spot-on analysis			
	log D_1	w_1	log D_2	w_2	D_1	w_1	D_2	w_2
All Traces	- 2.00	0.70	- 0.67	0.30	- 1.92	0.69	- 0.68	0.31
Bound Traces	- 2.07	0.85	- 1.33	0.14	- 2.04	0.78	- 1.16	0.22
Unbound Traces	- 2.08	0.62	- 0.67	0.38	- 2.04	0.54	- 0.86	0.46
Partial Traces	- 1.91	0.70	- 0.96	0.30	- 1.74	0.67	- 0.91	0.33

Supplementary Figure 10: Comparison of spot-on analysis and Gaussian mixture model fitting of single molecule traces. A) Frame-to-frame displacements of all single molecule traces and traces identified by correlative conventional and PALM imaging to be bound, unbound and partially bound are used for spot-on analysis to determine the diffusion coefficient of mobility populations and their weight fractions. **B)** Corresponding diffusion coefficient distributions from linear MSD fitting, two state Gaussian mixture model fits (dashed lines), sum of Gaussian fits (blue solid line) and diffusion coefficients of spot-on analysis (orange and yellow line). **C)** Diffusion coefficient and weight fraction comparison between two state Gaussian mixture model and spot-on analysis.

Supplemental Video 1: Representative PALM movie of MCP-HaloTag (PA-JF646 dye) with dCas9-GFP and 2xms2 telomere gRNA from correlative conventional PALM image acquisition sequence: This video represents the PALM signal acquired from a correlative conventional PALM movie of a cell transfected with MCP-HaloTag, dCas9-GFP, and 2xms2 telomere gRNA. This acquisition rate was 20 Hz. GFP and LED frames were removed from video. Video playback rate is 20 Hz.

Supplemental Video 2: Corresponding Conventional Movie of MCP-HaloTag (PA-JF646 dye) with dCas9-GFP and 2xms2 telomere gRNA: This video represents the conventional telomere signal for the PALM movie provided in supplemental video 1. Images were acquired every 10 frames at 20 Hz for a conventional frame acquisition rate of 2 Hz. Video playback rate is 2 Hz.

Supplemental Video 3: Conventional Movie of MCP-HaloTag (PA-JF646 dye) with dCas9-GFP and 2xms2 telomere gRNA: This video represents the conventional telomere signal of a correlative conventional PALM movie. Images were acquired every 10 frames at 20 Hz for a conventional frame acquisition rate of 2 Hz. Video playback rate is 2 Hz. This movie highlights the differences in telomere movements among different cells.

Supplemental Video 4: Conventional Movie of MCP-HaloTag (PA-JF646 dye) with dCas9-GFP and 2xms2 telomere gRNA: This video represents the conventional telomere signal of a correlative conventional PALM movie. Images were acquired every 10 frames at 20 Hz for a conventional frame acquisition rate of 2 Hz. Video playback rate is 2 Hz. This movie highlights the differences in telomere movements in among different cells

Supplemental Video 5: Representative PALM movie of MCP-HaloTag (PA-JF646 dye) with dCas9-GFP with no gRNA: This video represents the PALM signal acquired from a correlative conventional PALM movie of a cell transfected with MCP-HaloTag, dCas9-GFP, but no gRNA. This acquisition rate was 20 Hz. GFP and LED frames were removed from video. Video playback rate is 20 Hz.

Supplemental Video 6: Representative PALM movie of CMV-mEos2-NLS: This video represents the PALM signal acquired from a correlative conventional PALM movie of a cell transfected with CMV-mEos2-NLS, dCas9-GFP, but no gRNA. This acquisition rate was 20 Hz. GFP and LED frames were removed from video. Video playback rate is 20 Hz.

Supplemental References

1. Thompson,R.E., Larson,D.R. and Webb,W.W. (2002) Precise nanometer localization analysis for individual fluorescent probes. *Biophys J*, **82**, 2775–2783.

2. Nieuwenhuizen,R.P.J., Lidke,K.A., Bates,M., Puig,D.L., Grünwald,D., Stallinga,S. and Rieger,B. (2013) Measuring image resolution in optical nanoscopy. *Nat Methods*, **10**, 557–562.

3. Endesfelder,U., Malkusch,S., Fricke,F. and Heilemann,M. (2014) A simple method to estimate the average localization precision of a single-molecule localization microscopy experiment. *Histochem Cell Biol*, **141**, 629–638.
